# Supplementary material for: Emission impacts of China’s solid waste import ban and COVID-19 in the copper supply chain
Source: Nat Commun. 2021 Jun 18;12:3753. doi: 10.1038/s41467-021-23874-7 (PMC8213787; doi:10.1038/s41467-021-23874-7)
Supplement: Supplementary file 1 — Supplementary Information [file 41467_2021_23874_MOESM1_ESM.pdf]

Emission impacts of China's solid waste import ban and COVID-19 in the copper supply chain

Supplementary Information

John Ryter (ryterj@mit.edu)[1], Xinkai Fu (xinkaifu@mit.edu) [1], Karan Bhuwalka (bhuwalka@mit.edu) [2], Richard Roth (rroth@mit.edu) [2], Elsa Olivetti (elsao@mit.edu) [1, *]

[1] Department of Materials Science and Engineering, Massachusetts Institute of Technology, Cambridge, Massachusetts, United States of America
[2] Materials Systems Laboratory, Materials Research Laboratory, Massachusetts Institute of Technology, Cambridge, Massachusetts, United States of America
* Corresponding author

Contents

Emission impacts of China's solid waste import ban and COVID-19 in the copper supply chain 1
Supplementary Information 1
Supplementary Data: Abbreviations 2
Supplementary Methods: Regional Data and Evolution 3
Supplementary Methods: Semi-Fabricator Alloy Distribution Framework 7
Supplementary Methods: Scrap Price, Availability, and their Interplay 9
Supplementary Methods: Linear Programming Optimization Model 16
Supplementary Data: Import Data 20
Supplementary Methods: China's Concentrate Imports 21
Supplementary Data: Sensitivity to Scrap SD Elasticities 22
Supplementary Data: Figure Reproduction in Absolute Units 24
Supplementary Data: Remaining Environmental Impact Indicators 27
Supplementary Data: Impacts of Each Supply Chain Shock 39
Supplementary Data: Life Cycle Assessment Data 43
Supplementary Data: Compositional Information for Scrap, Products, Refined Materials 49
Supplementary Methods: Scenario Descriptions and Assumptions 50
Supplementary Methods: Near-Term System Response to COVID-19 Shocks 53
Supplementary Methods: Model Evolution Outline 55
References 57

Supplementary Data: Abbreviations

Supplementary Table 1. List of abbreviations used throughout this work, in alphabetical order

Phrase	Abbreviation	Phrase	Abbreviation
Autoregressive distributed lag	ARDL	Life cycle assessment	LCA
Capacity utilization	CU	London Metal Exchange	LME
Comparative ecological toxicity units	CTUe	Material flow analysis	MFA
Comparative human toxicity units	CTUh	Municipal solid waste	MSW
Construction and demolition	C&D	Net present value	NPV
Copper Development Association	CDA	Ordinary least squares	OLS
End of life	EOL	Ore grade elasticity (to total ore production)	OGE
End-of-life vehicles	ELV	Plate, sheet, strip	PSS
European Committee for Standardization	CEN	Rest of world	RoW
European Union	EU	Rod, bar, solids	RBS
Gross domestic product	GDP	Secondary ratio	SR
Industrial electrical waste	IEW	Shanghai Metals Market	SMM
Industrial non-electrical waste	INEW	Solvent extraction-electrowinning	SX-EW
Institute of Scrap Recycling Industries	ISRI	Supply and demand	SD
Internal rate of return	IRR	Total cash margin	TCM
International Copper Association	ICA	Treatment charges and refining charges	TCRC
International Copper Study Group	ICSG	Unified Numbering System	UNS
International Wrought Copper Council	IWCC	Waste electrical and electronic equipment	WEEE

Supplementary Methods: Regional Data and Evolution

Supplementary Table 2. Data sources and corresponding values collected.

International Copper Study Group (ICSG)[1,2]	Global average mine capacity utilization, total mining production, concentrate production, solvent extraction-electrowinning (SX-EW) production, primary and secondary smelting and refining production, refined copper consumption, direct melt copper consumption, regional distributions of global mining, refining, and semi-fabricator production
International Copper Association (ICA)[3]	Global sector to final product accounting matrix
Minsur	Global copper consumption by end-use sector
Glöser et al.[4]	Global sectoral lifetime distributions, consumption by final product to waste type accounting matrix, technical recycling efficiencies and collection rates by waste type, fabrication efficiencies by final product, sector to shape accounting matrix
International Wrought Copper Council (IWCC [5])	Sector to shape accounting matrix
Copper Development Association (CDA)[6-8]	Semi-fabricator shape and alloy data, general understanding of semi-fabricator system, scrap stream and scrap flows, energy consumption
CRU Group[9]	Annual TCRC
S&P Global Market Intelligence[10]	Individual (global and regional) minesite data, including head grades, total cash margin, annual production, etc.
Wood Mackenzie[11]	Individual (global and regional) refinery capacity utilization and primary and secondary refinery production; China concentrate, SX-EW, total mining, and primary and secondary refining production; China refined copper consumption and refined copper, scrap, and concentrate imports and exports
Shanghai Metals Market[12,13]	Base metal prices, China sectoral lifetimes, collection rates, and demand; China domestic and imported scrap supply, scrap demand
Fastmarkets AMM,[14] Fastmarkets MB[15]	Scrap and refined metal prices
UN Comtrade[16]	China refined copper consumption and refined copper, scrap, and concentrate imports

Historical data for China prior to data coverage was extrapolated from the existing data, assuming the average growth rate remained constant. Regionalization of primary and secondary refinery production mandated a reassessment of the dynamic panel regression models performed on the individual primary and secondary refinery production and capacity utilization data provided by Wood Mackenzie through 2016.[17] Refineries were separated into four groups – China and rest-of-world (RoW) primary and

secondary refineries, where primary refineries process only primary material and secondary refineries process a mixture of primary and secondary material – and aggregated to be treated as four representative refineries. For each individual refinery, the evolution of capacity utilization can be described by Supplementary Equation 1 while for each set of secondary refineries, the fraction of refinery production stemming from secondary material, the secondary ratio (SR), evolves according to Supplementary Equation 2.

$$log(CU_{i,t}) = \alpha + \rho log(CU_{i,t-1}) + \beta log(TCRC_t) + \mu_i + \varepsilon_{i,t} \tag{1}$$

$$log(SR_{i,t}) = \alpha + \rho log(SR_{i,t-1}) + \beta_1 log(TCRC_t) + \beta_2 \, log(Spread_{No.2,t}) + \mu_i + \varepsilon_{i,t} \tag{2}$$

Where $CU_{i,t}$ is the primary or secondary capacity utilization for each refinery i at time t, $TCRC_t$ is the global TCRC at time t as refinery-specific refining charges were unavailable, μ_i is the individual-invariant error term, and $\varepsilon_{i,t}$ is an idiosyncratic error. Given these equations are in log-linear form, each β represents the short-run TCRC or No.2 spread elasticity to CU or SR, while $\beta \div (1-\rho)$ represents the long-run elasticity, which accounts for the impact of past TCRC or No.2 spread values on CU or SR.[18,19] However, the coefficients for each of these parameters were not statistically significant at the regional level, and consequently the global values were used for each region. The resulting primary and secondary CU and SR evolution equations for the four representative refineries are shown in Supplementary Equations 3-S5.

$$CU_{primary,t} = CU_{primary,t-1} \cdot \left(\frac{TCRC_t}{TCRC_{t-1}}\right)^{0.0574} \tag{3}$$

$$CU_{secondary,t} = CU_{secondary,t-1} \cdot \left(\frac{TCRC_t}{TCRC_{t-1}}\right)^{0.153} \tag{4}$$

$$SR_t = SR_{t-1} \cdot \left(\frac{TCRC_t}{TCRC_{t-1}}\right)^{-0.197} \cdot \left(\frac{Spread_{No.2,t}}{Spread_{No.2,t-1}}\right)^{0.316} \qquad (5)$$

Regional initial CU values were calculated using the averages from individual China and RoW data, with initial capacity values calculated based on 2018 primary and secondary total production. Initial regional SRs were estimated by extrapolating average regional SRs from the average 2016 Wood Mackenzie value to 2018 using Supplementary Equation 5, and each of these parameters is shown in Supplementary Table 3.

Supplementary Table 3. Regional refinery hyperparameters for the simulation start time of 2018, where capacities are reported in kilotonnes (kt, thousand metric tonnes), CU and SR are fractions of 1.

	China	Rest of World
Primary capacity	6,650 kt	818 kt
Primary CU	0.875	0.854
Primary CU long-run elasticity to TCRC	0.0574	0.0574
Secondary Capacity	4,240 kt	11,200 kt
Secondary CU	0.886	0.836
Secondary CU long-run elasticity to TCRC	0.153	0.153
SR	0.420	0.293
SR long-run elasticity to TCRC	-0.197	-0.197
SR long-run elasticity to No.2 scrap spread from cathode price	0.316	0.316

Supplementary Methods: Semi-Fabricator Alloy Distribution Framework

The regional, sectoral distribution of total copper demand was obtained using exogenous economic growth indicators and copper use intensity evolution, as described in the Methods section of this work and by Dr. Xinkai Fu.[18] Sectors are construction, electrical, transport, industrial, and other, while regions are China, the European Union, Japan, North America, and other. Following regrouping to match the China-RoW division used in this model, a sector-shape conversion matrix was used to convert sectoral consumption into two unalloyed shapes – wire and other – and five unalloyed shapes – wire, tube, RBS (rods, bars, and solids), PSS (plates, sheets, and strips), and castings. This static matrix was derived by grouping sector and shape data reported by Glöser et al and the International World Copper Council, using the average distribution of the last four years' reported data, and is shown in Supplementary Table 4.[4,5] Due to a lack of data availability, it was assumed that this distribution holds constant between regions as well.

Supplementary Table 4. The sector-shape conversion matrix

		Construction	Electrical	Industrial	Transport	Other
Unalloyed	Wire	0.451	0.944	0.361	0.569	0.480
	Other	0.407	0.002	0.136	0.139	0.252
Alloyed	Tube	0.003	0.035	0.055	0.013	0.007
	RBS	0.100	0.019	0.260	0.034	0.036
	PSS	0.001	0.000	0.024	0.110	0.150
	Wire	0.001	0.000	0.016	0.006	0.039
	Cast	0.037	0.000	0.148	0.129	0.036
Total:		1.000	1.000	1.000	1.000	1.000

The resulting regional copper demand by shape was further broken down into alloy designations to permit a compositional distribution of copper demand for the linear programming optimization model. Unalloyed shapes were assumed to have the same high-copper composition of the CW003A CEN alloy or similar, while alloyed shapes required a more thorough treatment.

For each alloy, some specific scrap grades are preferred, particularly those with similar chemical compositions to the alloy. Therefore, the breakdown of direct melt scrap consumption depends on the grades and quantities of alloys produced. Mathematically, consumption by alloy grade can be calculated as shown in Supplementary Equation 6.

$$C_{a_i,t} = \sum_{s_j \epsilon s} C_{s_j,t} A_{s_j,a_i} \tag{6}$$

$C_{a_i,t}$ represents consumption of alloy grade a_i at year t, and A_{s_j,a_i} is an accounting matrix to calculate the fraction of alloy a consumed for each shape s_j. The sum is over s, a set containing the five alloyed semis shapes (tube, RBS, PSS, wire, and castings). Because no reported data is readily available for the accounting matrix A_{s_j,a_i}, the fraction of each shape comprised by each of the 190 alloyed semis was determined based on the CDA's copper alloy supplier database.[6] This database provided alloy supplier counts for each semis shape, and while it was limited to suppliers in the United States, it was assumed that average sectoral alloy compositions within each shape would be sufficiently similar between regions. Based on industry interviews, it was further assumed that the 80-20 rule would apply, which states that 80% of a market is dominated by 20% of its constituents.

Here, the rule was adjusted slightly to account for the large number of alloys with either no suppliers listed by the CDA or with a small number of suppliers to represent the large number of specialty alloys that introduce trace amounts of less common elements to the eventual scrap stream. This large quantity of alloys with few or no suppliers produced a highly-skewed distribution in the number of suppliers. As

such, alloys were broken into groups representing high-demand, moderate-demand, and specialty alloys comprising 70%, 20%, and 10% of a given shape's production, respectively. High-demand alloys had a number of suppliers greater than two standard deviations above the mean number of suppliers, moderate-demand alloys had a number of suppliers greater than half one standard deviation above the mean number of suppliers, and specialty alloys were all others. Each alloy's production fraction is then equal to the group fraction multiplied by the alloy's supplier fraction within its group. The accounting matrix A_{s_j,a_i} can then be represented by Supplementary Equation 7 below.

$$A_{s_j,a_i} = \frac{0.7 n_{s_j,a_i} \delta\left(n_{s_j,a_i} > mean\left(n_{s_j}\right) + 2stdev\left(n_{s_j}\right)\right)}{\sum_{a_i \in a} n_{s_j,a_i} \delta\left(n_{s_j,a_i} > mean\left(n_{s_j}\right) + 2stdev\left(n_{s_j}\right)\right)} + \frac{0.2 n_{s_j,a_i} \delta\left(n_{s_j,a_i} > mean\left(n_{s_j}\right) + 0.5stdev\left(n_{s_j}\right) \cup n_{s_j,a_i} < mean\left(n_{s_j}\right) + 2stdev\left(n_{s_j}\right)\right)}{\sum_{a_i \in a} n_{s_j,a_i} \delta\left(n_{s_j,a_i} > mean\left(n_{s_j}\right) + 0.5stdev\left(n_{s_j}\right) \cup n_{s_j,a_i} < mean\left(n_{s_j}\right) + 2stdev\left(n_{s_j}\right)\right)} + \frac{0.1 n_{s_j,a_i} \delta\left(n_{s_j,a_i} < mean\left(n_{s_j}\right) + 0.5stdev\left(n_{s_j}\right)\right)}{\sum_{a_i \in a} n_{s_j,a_i} \delta\left(n_{s_j,a_i} < mean\left(n_{s_j}\right) + 0.5stdev\left(n_{s_j}\right)\right)} \quad (7)$$

Where A_{s_j,a_i} is the accounting matrix from above for shape s_j and alloy a_i, n_{s_j,a_i} is the number of suppliers for each alloy within each shape, δ is the Dirac delta function, and the mean and standard deviation are calculated using all alloys within a given shape. Alloys with no suppliers listed were given A_{s_j,a_i} of 0.005, and the accounting matrix was then normalized such that the sum over alloys for each shape was equal to one. The accounting matrix was then perturbed and renormalized iteratively until the average copper content of each alloyed semis shape matched that from ICA data. The accounting matrix is then assumed to remain static with time.

Supplementary Methods: Scrap Price, Availability, and their Interplay

Scrap markets were assumed to be sufficiently illiquid that purchasing followed an order book formulation, where increasing consumption of any scrap grade increases the average price of that grade for the market and the resulting average scrap price is a function of availability. For example, if a

fabricator needs 100 kt of scrap and the cheapest available scrap is 50 kt at US$5000 per tonne, with an additional 100 kt is available at US$6000 per tonne, the average price of scrap for that fabricator will then be US$5500 per tonne, as shown in Supplementary Equation 8. Using China copper scrap import transaction-level data for 2018 from Big Trade Data, we found empirical evidence for order book behavior (Supplementary Figure 1a). Order book behavior means that raw materials are available at a variety of prices, meaning that the total price of a given purchase must be represented by Supplementary Equation 8.

$$Average\ Cost = \sum_{u \in unit\ costs} u * q_u \tag{8}$$

Where u represents an individual unit cost within the set of unit costs corresponding with the set of quantities q, with q_u being the quantity purchased at a given unit cost. For the majority of scrap grades represented in this data, the characteristic average cost curve (Supplementary Figure 1b) was produced, with the average value of this curve approximately equal to the year's scrap price. Assuming that all scrap grades would follow similar curves, we developed a formulation for expressing this curve in piecewise linear form (Supplementary Figure 1b, Supplementary Equation 9) as a function of scrap price, availability, and quantity demanded. This curve was based on parameters for No.1 copper scrap because it had the greatest quantity of available data, producing the coefficients in Supplementary Equation 9 from a piecewise linear best fit of the data shown in Supplementary Figure 1b, then scaling such that the curve average unit price matched the global average unit price for 2018.

$$Market\ Average\ Cost(x) = \begin{cases} 0, & x = 0 \\ 0.777p + 0.782\frac{p}{a}x, & 0 < x < 0.187a \\ 0.872p + 0.276\frac{p}{a}x, & 0.187a \le x < 0.812a \\ 0.672p + 0.522\frac{p}{a}x, & 0.812a \le x < 1 \end{cases} \tag{9}$$

Where we calculate the average cost of a given scrap grade for the entire market based on the quantity demanded by the market x, the quantity of that scrap grade available a, and the reported or calculated market average unit price for that scrap grade p.

Supplementary Figure 1: Order book formulation for No.2 scrap

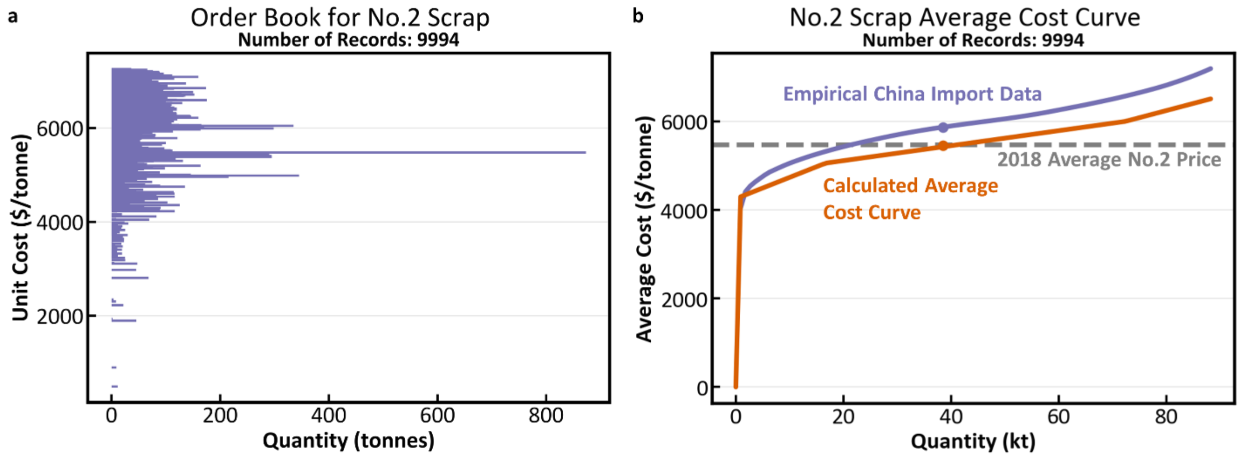

a, Reported China copper scrap import transaction quantities and unit costs for the 9994 records identified as corresponding to the No.2 scrap grade in 2018. **b**, The resulting average cost curves from the empirical China import transaction data calculated using Supplementary Equation 8 (blue) and the calculated piecewise linear average cost curve using Supplementary Equation 9, the quantity of available scrap, and the 2018 reported No.2 price. Dots indicate the average unit price of the corresponding dataset. Underlying data used to create this figure may be found in a data repository at https://doi.org/10.6084/m9.figshare.14390489.v1.[20]

Within the linear programming optimization model described in Supplementary Methods: Linear Programming Optimization Model, the total cost of the market's consumption of a given scrap grade is calculated by integrating Supplementary Equation 9, while for refined materials it is simply the product of the quantity demanded and the unit cost. These values were used to create piecewise linear penalty functions for each refined metal and scrap grade such that the total price was minimized. Post-industrial recycled material prices were calculated as a constant fraction of refined copper price, reflecting the expectation that nearly 100% of new scrap generated each year is consumed due to its capacity for direct melt consumption.

Historical scrap prices were reported by Fastmarkets MB and the American Metal Market, and average scrap price and refined metal price evolution are calculated as follows. A given year's scrap and cathode prices are calculated using the supply-demand balance, or change in inventory, produced by historical data or model evolution in the year prior using Supplementary Equations 10 and S11, and the elasticity values shown in Supplementary Table 4. Prices for non-copper refined metals were assumed to evolve proportionally to refined copper, as shown in Supplementary Equation 12.

$$P_{cathode,t} = P_{cathode,t-1} + \beta_{cathode\ SD} * Bal_{cathode} \tag{10}$$

$$Spread_{scrap,t} = Spread_{scrap,t-1} + \beta_{scrap\ SD} * Bal_{scrap} + \beta_{scrap\ cathode} * (P_{cathode,t} - P_{cathode,t-1}) \tag{11}$$

$$P_{metal,t} = P_{metal,t-1} * \frac{P_{cathode,t}}{P_{cathode,t-1}} \tag{12}$$

Where P represents the price of copper cathode or that of the other refined metals considered in this study (Al, Fe, Mn, Ni, Pb, Sn, Zn) based on subscript, spread is the term used for the difference between cathode price and scrap price within a given time period, and the β terms are explained and given values in Supplementary Table 5. Supplementary Equations 11 and S12 are evaluated for each scrap grade and refined metal, respectively, where each of the alloyed scrap grades considered here (aluminum bronze, cartridge, manganese bronze, nickel silver, ocean, red brass, leaded red brass, tin bronze, leaded tin bronze, yellow brass, and leaded yellow brass) is evaluated using its own price but the same alloyed elasticities.

Supplementary Table 5. List of symbols for use in Supplementary Equations 10-S12, where SD refers to supply and demand.

Symbol	Name	Value
$\beta_{cathode\ SD}$	Cathode SD elasticity	-0.645598
$\beta_{scrap\ SD}$	No.2 spread SD elasticity	0.0845
	No.1 spread SD elasticity	0.02955
	Alloyed scrap spread SD elasticity	0.193818
$\beta_{scrap\ cathode}$	No.2 spread cathode elasticity	0.18399
	No.1 spread cathode elasticity	0.064346
	Alloyed scrap spread cathode elasticity	0.422019

We now present the data used for calculating scrap generation at the level of detail required for use in the semi-fabricator alloy distribution framework described in Supplementary Methods: Semi-Fabricator Alloy Distribution Framework, where a lognormal distribution of material lifetimes was used to calculate scrap generation, recycling collection and recovery rates were used to calculate old scrap entering useable inventories, and fabrication efficiencies, recovery rates, and external scrap ratios were used to calculate new scrap entering useable inventories. Lifetimes, fabrication efficiencies, and new scrap generation rates are shown in Supplementary Table 6; regional scrap collection and recovery rates are shown in Supplementary Table 7. The new scrap generation rate is equal to $\frac{1}{fabrication\ efficiency} - 1$.

Supplementary Table 6. Sectoral lifetimes, fabrication efficiencies, and new scrap generation rates for China and RoW.

Sector	RoW Lifetime (Years)	RoW Fabrication efficiency	RoW New scrap generation rate	CN Lifetime (Years)	CN Fabrication efficiency	CN New scrap generation rate
Plumbing	40	0.95	0.05	28	0.95	0.05
Building Plant	40	0.9	0.11	28	0.95	0.05
Architecture	50	0.85	0.18	28	0.95	0.05
Communications	30	0.9	0.11	28	0.95	0.05
Electrical Power	40	0.9	0.11	25	0.93	0.08
Telecommunications	30	0.9	0.11	28	0.95	0.05
Power Utility	30	0.85	0.18	25	0.93	0.08
Electrical Industrial	15	0.8	0.25	15	0.95	0.05
Non-Elec. Industrial	20	0.9	0.11	15	0.95	0.05
Electrical Automotive	12	0.75	0.33	12	0.75	0.33
Non-Elec. Automotive	15	0.9	0.11	13	0.97	0.04
Other Transport	25	0.8	0.25	13	0.97	0.04
Consumer	8	0.75	0.33	11	0.96	0.04
Cooling	10	0.8	0.25	11	0.96	0.04
Electronic	5	0.75	0.33	4.9	0.95	0.05
Diverse	10	0.75	0.33	10	0.75	0.33

Supplementary Table 7. Regional end-of-life (EOL) scrap collection and recovery rates for the five waste categories considered in this study: construction and demolition (C&D), municipal solid waste (MSW), waste electrical and electronic equipment (WEEE), end-of-life vehicles (ELV), industrial electrical waste (IEW), and industrial non-electrical waste (INEW).

Waste Category	RoW EOL collection rate	RoW Recovery rate	CN EOL collection rate	CN Recovery rate
C&D	0.72	0.9	0.75	0.9
MSW	0.05	0.55	0.1	0.6
WEEE	0.63	0.55	0.3	0.6
ELV	0.91	0.7	0.52	0.8
IEW	0.66	0.75	0.5	0.98
INEW	0.68	0.2	0.7	0.88

Based on interviews with industry experts, we assumed the external scrap ratio to be 0.1, with home scrap and exchange scrap accounting for the remainder of new scrap generation. External scrap is that which manufacturers sell to scrap dealers or processors. Home scrap, also called in-house scrap or runaround scrap, is both generated and directly remelted for consumption at the manufacturer. Because it never leaves the fabrication facility, home scrap is not included in consumption statistics and its generation is highly uncertain. Exchange scrap, also called toll scrap, is incapable of direct remelt at the manufacturer and is instead sent to upstream scrap processors, processed, and returned to the manufacturer. Historical new scrap consumption is calculated based on total scrap consumption and the calculated old scrap generation. Based on fabrication efficiencies, the total quantity of pre-consumer scrap is known; by incorporating the calculated new scrap consumption, we then calculated the fraction

of home and exchange pre-consumer scrap, which has evolved over time as shown in Supplementary Figure 2. Throughout this study, we refer to the sum of external and exchange scrap as new scrap or post-industrial scrap. For data beyond 2018, we assumed increases in the home scrap ratio based on improving technology.

Supplementary Figure 2: Home, exchange, and external scrap ratios for manufacturers

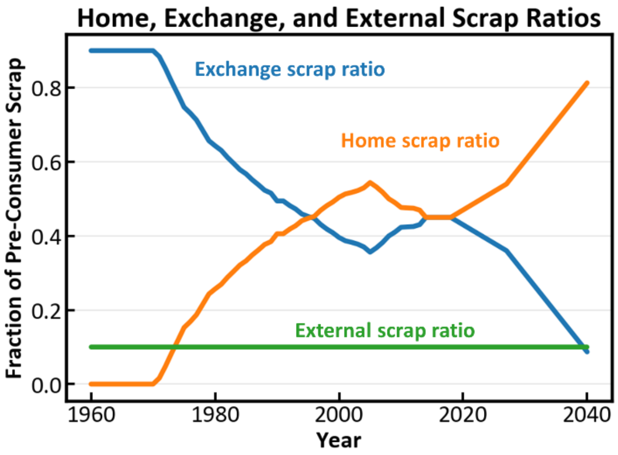

Home, exchange, and external scrap ratios for scrap generated by manufacturers. Underlying data used to create this figure may be found in a data repository at https://doi.org/10.6084/m9.figshare.14390489.v1.

Supplementary Methods: Linear Programming Optimization Model

Consumption for each of the 190 alloys can be calculated based on Supplementary Equation 6. Refined metal and direct melt scrap consumption are then determined by the linear programming optimization model within the blending module. The major model assumptions are:

1. Alloyed semis (brass mills and foundries) have the capability to directly melt and blend different raw materials including refined metals and grades of alloyed copper scraps into alloys. It is assumed that there is no loss of materials during the blending process, and the chemical composition of the final blended product is the weighted average of all raw materials.

2. The only quality constraint on the blended alloy product is the compositional specifications of elements in the alloy. In reality, there could be other quality constraints in the fabrication processes, such as requirements for mechanical properties and durability that might prohibit specific scrap grades into entering the melt. These other constraints are essentially neglected in this model.
3. It is assumed that raw material costs only come from scraps and refined metals purchased. Other costs such as energy, labor, fixed costs, etc. are considered as constant. As long as compositional requirements are met, fabricators seek to minimize raw material costs by purchasing the cheapest mix of raw materials possible.
4. Fabricators allow for the possibility that some products could be outside compositional requirements. Rather, they control for the success rate, or the fraction of products that meet compositional requirements.
5. All refined raw materials are assumed to be infinitely available.
6. The quantity of refined copper consumed globally each year is determined outside the blending module, under the assumption that its liquidity enables immediate redistribution between China and RoW.
7. Secondary refinery scrap consumption is primarily composed of No.2 scrap, though secondary refineries may also use up to 5% of alloyed scrap available.

Based on these assumptions, a blending optimization model is formulated mathematically as follows:

Objective:

$$\min(RMC) = \sum_i M_i P_i \tag{13}$$

Subject to:

1. Compositional constraints:

$$\forall j, Pr\{\sum_i M_i X_{ij} \leq c_j^U Q\} > \lambda \tag{14}$$

$$\forall j, Pr\{\sum_i M_i X_{ij} \geq c_j^L Q\} > \lambda \tag{15}$$

2. Mass balance:

$$\sum_i M_i = Q \tag{16}$$

The description of each variable/symbol can be found in Supplementary Table 7. This model uses 214 grades of raw materials, including 8 refined metals, 2 grades of unalloyed scrap (No.1, ISRI trade name barley and No.2, ISRI grade birch), 13 grades of alloyed scrap, and 191 categories of post-industrial recycled material. The complete list of these grades and their compositional ranges are shown in Supplementary Table 21. Due to limited data availability for both scrap compositions and scrap prices, the 15 scrap grades used here represent groups of scrap grades rather than specific ISRI grades. For example, the nickel-silver scrap grade in this model corresponds to six ISRI grades (Maize, Major, Malar, Malic, Naggy, Niece). A chance constrained formulation is used here, where the compositional constraints are probabilistic instead of deterministic. This formulation corresponds to assumption 4 mentioned above. In order to find the optimal solution, the compositional constraints are represented through the fuzzy number approach, following what is used in a previous study.[21] The total cost of the market's consumption of a given scrap grade is calculated by integrating Supplementary Equation 9 described in Supplementary Methods: Scrap Price, Availability, and their Interplay; while for refined materials this value is simply the product of the quantity demanded and the unit cost. These values were used to create piecewise linear penalty functions for each refined metal and scrap grade within each region, which enabled market-wide price minimization.

Supplementary Table 8. Description of variables used in the blending optimization model

Symbol	Description
RMC	Total raw material cost
M_i	Mass of raw material grade i consumed
P_i	Price of raw material grade i
X_{ij}	Mass fraction of element j in material i
$c_j^{U/L}$	Upper/lower limit for element j in alloy based on its specification
Q	Mass of alloy produced
λ	Confidence level of product meeting specification

The direct melt scrap breakdown model described above takes regional fabricator consumption, raw material prices, and global refined copper demand as input, and calculates the amount of direct melt scrap consumption for each scrap grade, refined metal, and distribution of these quantities between China and RoW. Given that global refined copper demand is calculated endogenously as a function of cathode price outside the blending model, this model was not permitted the additional degree of freedom allotted by determining global refined copper demand, and therefore refined copper demand acted as an additional constraint.

Supplementary Data: Import Data

Supplementary Table 9. Year over year change in copper scrap exports to China and change in imports for the nations addressed in this study, in kt copper content. Blank cells indicate a lack of data availability.

	kt change in exports to China			kt change in imports		
	2017-2018	2018-2019	2017-2019	2017-2018	2018-2019	2017-2019
China	0.0	0.0	0.0	-49.0	-19.1	-68.0
Rep. of Korea	3.8	12.6	16.4	31.8	-1.2	30.6
India	0.2	4.3	4.4	9.0	13.5	22.4
Germany	-16.2	-6.8	-23.0	39.2	-2.1	37.1
Taiwan	17.6	38.3	55.9	11.0	38.3	55.9
Belgium	6.9	-11.2	-4.3	10.4	18.0	28.4
Malaysia	13.0	201.1	214.0	50.6	20.0	70.6
Canada	7.5	7.2	14.7	10.2	16.9	27.1
Viet Nam	1.8	1.4	3.2	-0.5	2.5	2.1
Indonesia	5.4	15.6	20.9	12.3	7.7	20.0
USA	-41.7	-103.1	-144.8	-43.8	7.0	-36.8
Japan	38.0	-22.1	15.8	23.5	-40.3	-16.8
Pakistan	1.1	-2.7	-1.6	8.2	6.9	15.0
Singapore	5.6	14.9	20.5	0.4	-2.8	-2.5
Thailand	-41.2	25.8	-15.4	-9.2	15.0	5.8
United Arab Emirates	1.8	25.7	27.5	-11.3	0.0	-11.3
United Kingdom	13.3	12.9	26.3			
EU	-7.9	-24.6	-32.5			

Supplementary Table 10. The percentage of each country's copper scrap exports that go to China in each year and the year over year change in the copper content (%) for copper scrap imports for each country, where the percentage given is relative to the initial year in the range displayed. Blank cells indicate a gap in data availability.

	% of country's exports going to China			Year over year % change in %Cu of copper scrap imports		
	2017	2018	2019	2017-2018	2018-2019	2017-2019
China				42.5	40.1	99.6
Rep. of Korea	89.5	67.9	38.3	8	-4.1	3.6
India	5.7	4.9	29.8	-4	-11	-14.5
Germany	36.7	24.3	14.7	3.3	0.7	4.0
Taiwan	51.4	49.2		2.7		
Belgium	39.7	20.9	15.3	2.2	2.8	5.1
Malaysia	8.7	44.3	81.4	-44.3	9.1	-39.3
Canada	38.1	23.3	19.1	5	-2.9	2.0
Viet Nam	5.2	6.4	5.5	3.1	-0.4	2.7
Indonesia	43.7	34.7	25.8	3.3	-13.4	-10.5
USA	68.4	29.6	10.1			
Japan	93.5	80.4	39.7	4	-4.3	-0.5
Pakistan	29.6	17.4	1.1	-78	-84.6	-96.6
Singapore	0.0	33.0	33.4		-8.5	
Thailand	26.3	32.4	31.1	17.4	5.1	23.5
United Kingdom	49.5	41.8	25.3			
United Arab Emirates	42.0	38.5		61.1		

Supplementary Methods: China's Concentrate Imports

China's concentrate imports were calculated using values calculated using primary refining production in China (divided by 0.99, the concentrate to cathode efficiency assumed in this model) and subtracting the estimated concentrate production from mining within China, where China's share of global concentrate production was assumed to hold constant at the 2017 value of 9% reported by ICSG.[1]

Supplementary Figure 3: China's historical and simulated concentrate imports

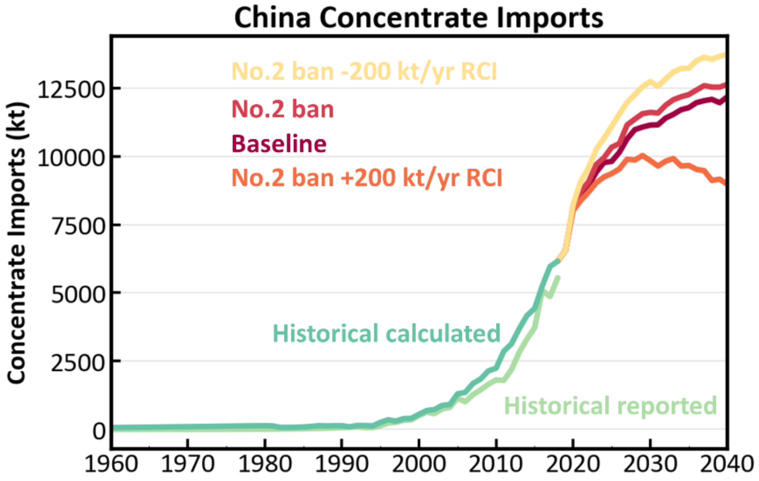

China's historical and simulated concentrate imports under baseline and No.2 ban scenarios, with or without ±200 kt/yr changes in refined copper imports (RCI). Underlying data used to create this figure may be found in a data repository at https://doi.org/10.6084/m9.figshare.14390489.v1.

Supplementary Data: Sensitivity to Scrap SD Elasticities

One of the key factors in determining the distributions of scrap and refined copper consumption between China and RoW was the evolution of scrap spreads, calculated as shown in Supplementary Methods: Scrap Price, Availability, and their Interplay. As such, we conducted a sensitivity analysis for the impact of scrap spread elasticity to scrap supply-demand balance. In this set of scenarios, we halved or doubled the elasticity, demonstrating that increasing its value produces a small increase in the CO_2e emissions response due to China's more rapid shift away from using scrap material as its price rises in conjunction with the solid waste import ban, even as the RoW's response enables a small decrease primary refining and increase in secondary refining. Decreasing the scrap spread SD elasticity produces the opposite effect.

Supplementary Figure 4: Global and regional system response distributions for varying scrap SD elasticity

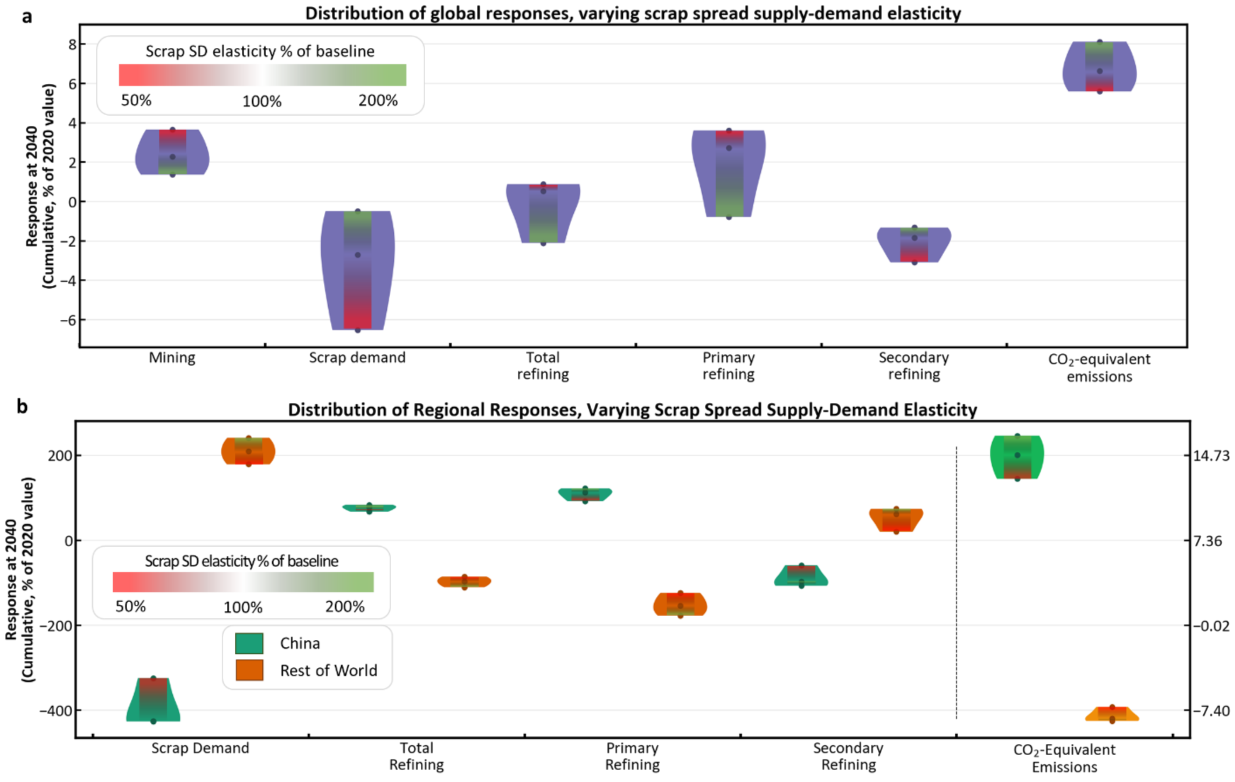

a, Violin plot showing the distributions of global responses for varying scrap spread elasticity to the supply-demand balance. **b**, Violin plot showing the distributions of regional responses for each supply chain actor, with CO2e emissions highlighted as a system-level response and plotted on a secondary axis. China is shown in green, the rest of world in orange. Underlying data used to create this figure may be found in a data repository at https://doi.org/10.6084/m9.figshare.14390489.v1.

Supplementary Data: Figure Reproduction in Absolute Units

Supplementary Figure 5: Results of China's solid waste import ban

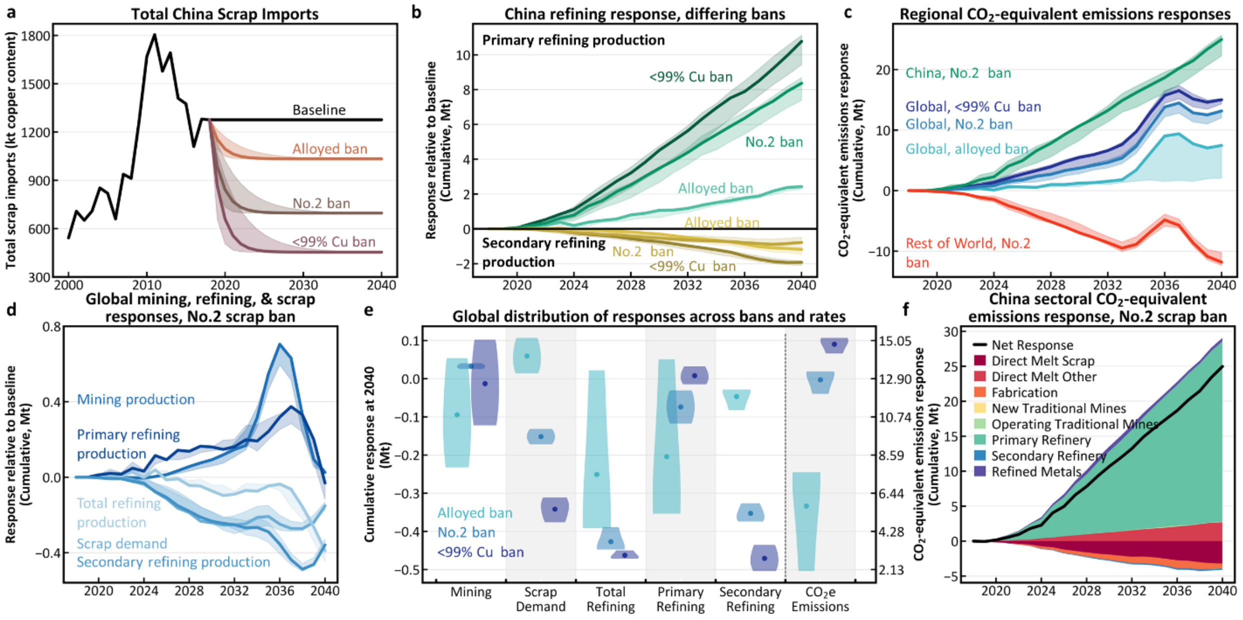

Reproduction of Figure 2 (main text) using cumulative departure from baseline in million metric tonnes as y-axis units for each plot. a, Scrap imports for each scenario in copper content. b, Primary and secondary refining responses in China relative to baseline for each scenario. c, Regional CO_2e emissions responses for each scenario at the global level and regional results for the No.2 scrap ban over the simulation period. d, Cumulative global mining, refining, and scrap demand responses for the No.2 scrap ban over the simulation period. e, The distributions of global mining, scrap demand, total refining, primary refining, secondary refining, and CO_2e emissions responses for each ban, evaluated cumulatively at 2040 relative to baseline. Points represent the mean of the three ban rates, while the shaded regions represent the distributions of ban rate results. CO_2e emissions are plotted on a secondary axis. f, Cumulative sectoral CO_2e emissions response for China, where all increasing impacts were plotted above the x-axis, all decreasing impacts were plotted below the x-axis, and the black line represents the net response within China as a result of these sectoral changes. Underlying data used to create this figure may be found in a data repository at https://doi.org/10.6084/m9.figshare.14390489.v1.

Supplementary Figure 6: Results for changing China's refined copper imports coincident with No.2 ban

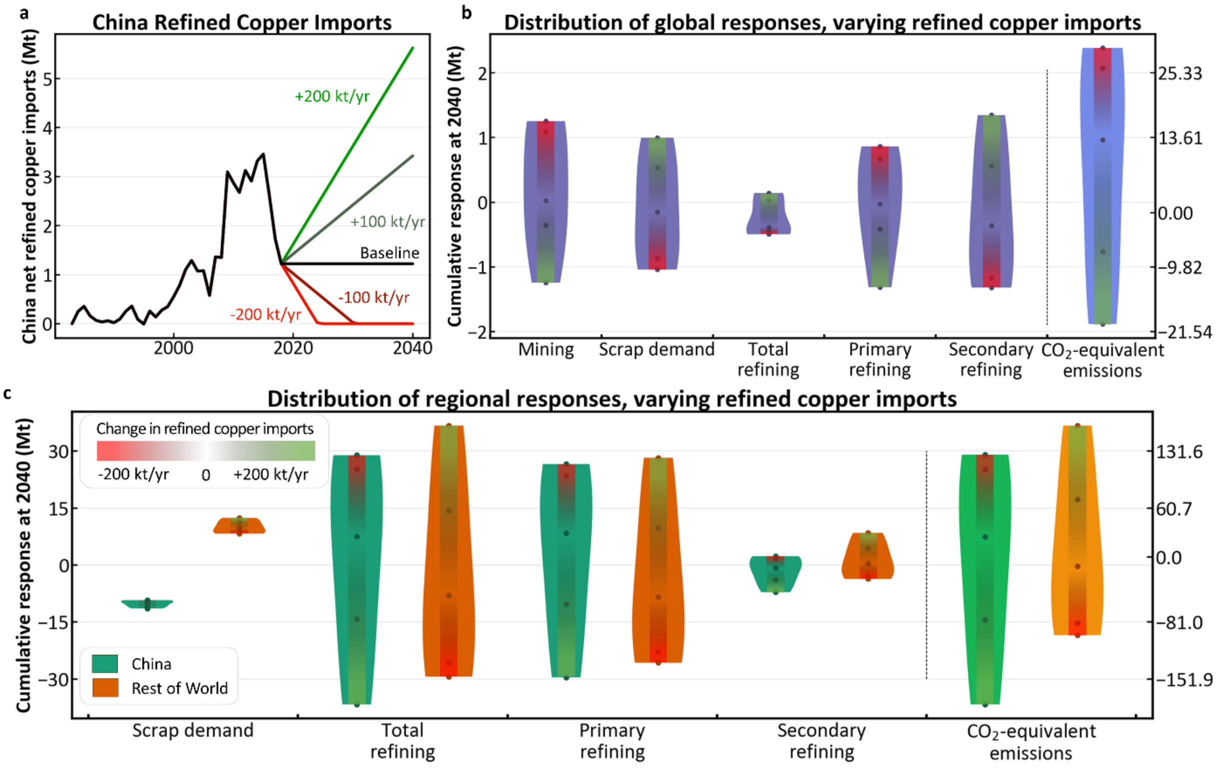

Reproduction of Figure 3 (main text) using cumulative departure from baseline in million metric tonnes as y-axis units for each plot. **a**, Historical Chinese net refined copper imports and scenario definition, where in this case the baseline corresponds with the No.2 scrap ban and refined copper imports are increased or decreased at rates of 100 or 200 kt/year, with minimum zero. **b**, Violin plot showing the distributions of global responses for varying refined copper imports, where CO2e emissions are highlighted as an aggregate response and are plotted on a secondary axis. **c**, Violin plot showing the distributions of regional responses for each supply chain actor, with CO2e emissions highlighted as a system-level response and plotted on a secondary axis. China is shown in green, the rest of world in orange. Underlying data used to create this figure may be found in a data repository at https://doi.org/10.6084/m9.figshare.14390489.v1.

Supplementary Figure 7: System response to COVID-19 scenarios and supply chain disruption sensitivities

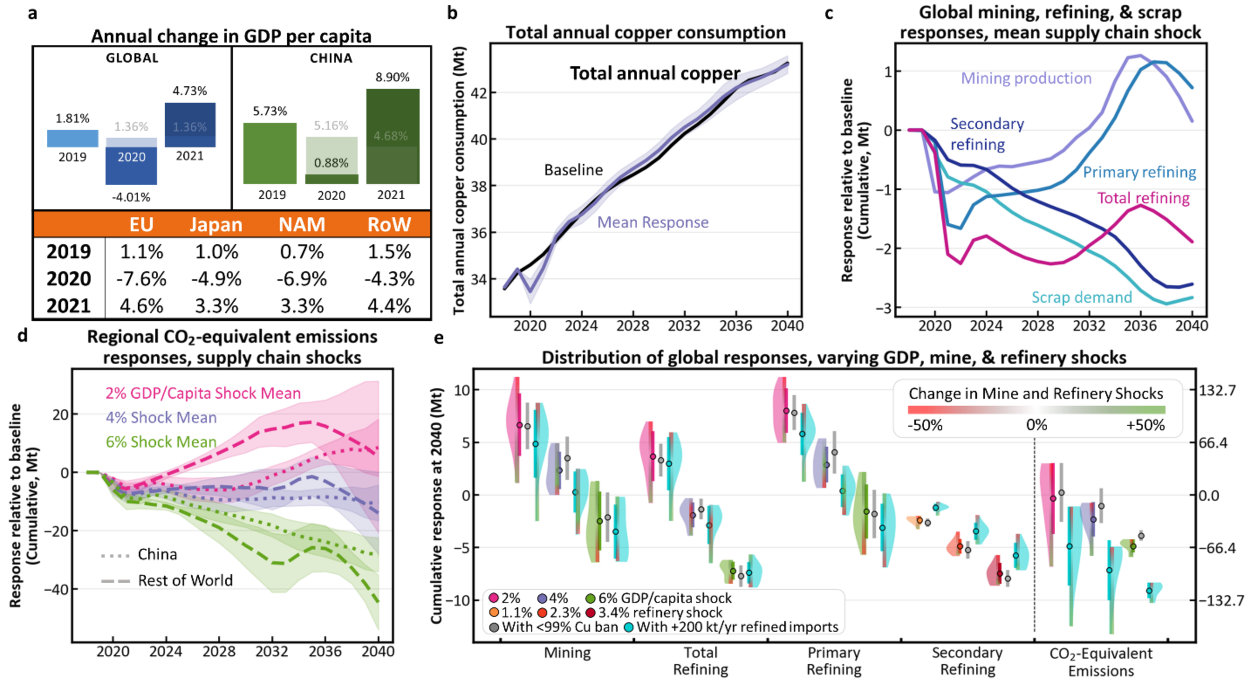

a, The annual changes in GDP per capita for 2019, 2020, and 2021 used in the COVID-19 response scenario, using values adapted from annual change in GDP from the International Monetary Fund. Baseline values for China and global are shown in gray. **b**, Global annual copper consumption including alloyed and unalloyed refined and scrap copper consumption for baseline and the mean COVID-19 scenario response. Shaded areas represent one standard deviation. **c**, Cumulative global secondary refining, scrap demand, mining production, total refining, and primary refining responses relative to baseline as a percent of the 2020 value, labelled from top to bottom using 2040 as reference. Standard deviations not shown for clarity. **d**, Cumulative CO_2e emissions responses relative to baseline for China and RoW as a percent of the 2020 global value. **e**, Violin plot showing the distribution of global responses for COVID-19 response scenarios using 2, 4, and 6% declines in global GDP per capita from 2019-2020, with mean values shown as same-colored points. Gray points and bars represent the mean system response and standard deviation when the No.2 scrap China solid waste import ban is simulated simultaneous with the COVID-19 shocks. Green and red bars indicate the magnitude of the mine and refinery system shocks. For secondary refining, GDP changes produced near-equal violin plots and here the data are grouped by the three levels of refinery shock

instead. Underlying data used to create this figure may be found in a data repository at https://doi.org/10.6084/m9.figshare.14390489.v1.

Supplementary Data: Remaining Environmental Impact Indicators

Supplementary Figure 8: Environmental impact responses broken down by supply chain actor, relative to baseline

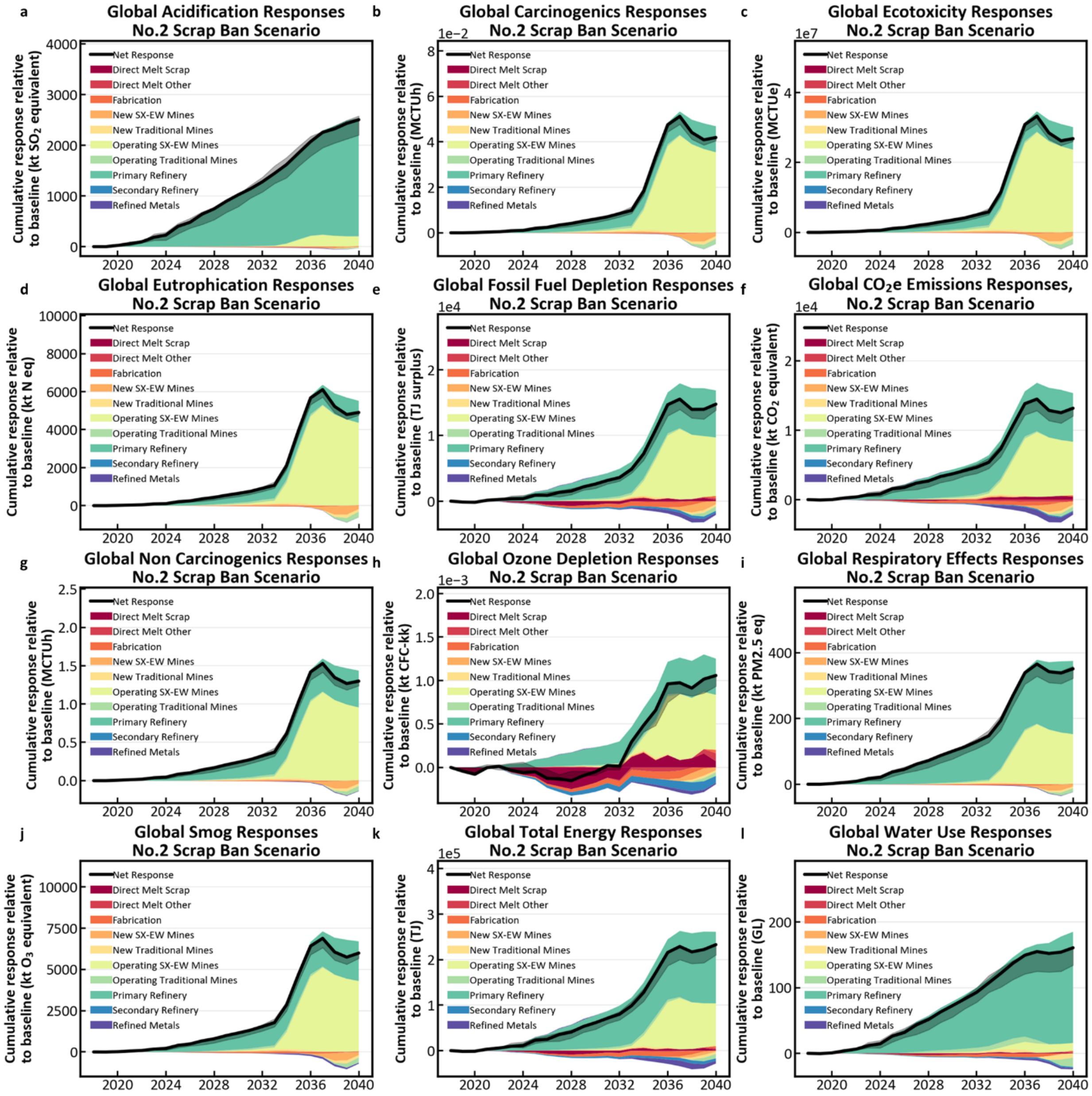

All responses are reported relative to baseline in their original calculated units, and gray shading around the net response represents ban rate variation. **a**, Global CO_2e emissions response. **b**, Global SO_2e emissions response. **c**,

Global carcinogenics human health (disease cases) impact response. **d**, Global ecotoxicity (fraction of potentially-affected species integrated over time and volume of freshwater compartment) response. **e**, Global N emissions contributing toward eutrophication. **f**, Global fossil fuel depletion response. **g**, Global non-carcinogenics human health (disease cases) impact response. **h**, Global ozone depletion impact response. **i**, Global respiratory effects (particulate matter less than 2.5 micrometers in diameter) impact response. **j**, Global smog (O_3 emissions) impact response. **k**, Global total energy consumption response. **l**, Global water use response. Underlying data used to create this figure may be found in a data repository at https://doi.org/10.6084/m9.figshare.14390489.v1**.**

Supplementary Figure 9: Environmental impact responses broken down by supply chain actor, as % of 2020 value

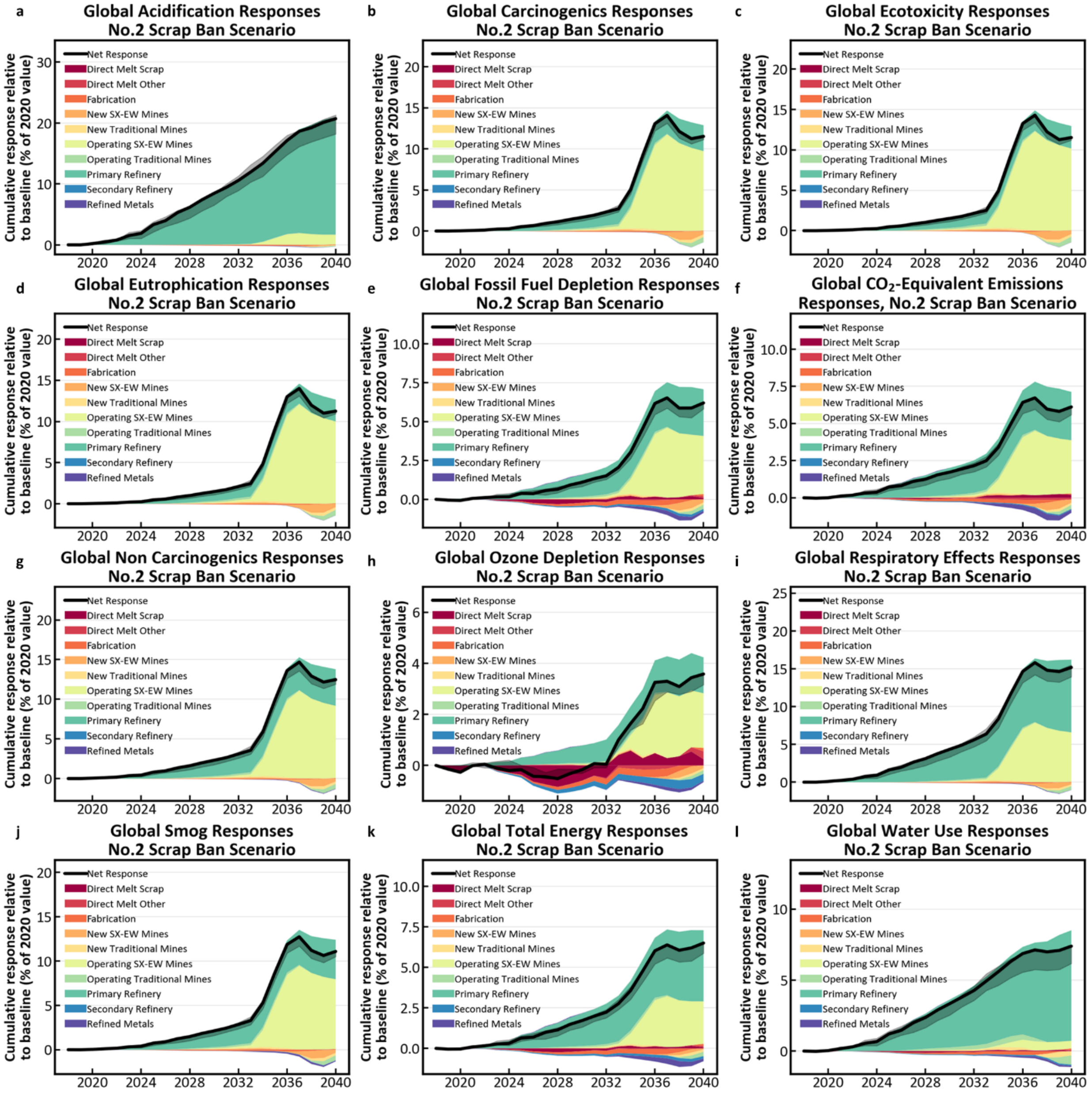

All responses are reported relative to baseline as a percent of the 2020 value, and gray shading around the net response represents ban rate variation. **a**, Global SO_2e emissions response. **b**, Global carcinogenics human health (disease cases) impact response. **c**, Global ecotoxicity (fraction of potentially-affected species integrated over time and volume of freshwater compartment) response. **d**, Global N emissions contributing toward eutrophication. **e**, Global fossil fuel depletion response. **f**, Global CO_2e emissions response. **g**, Global non-carcinogenics human health

(disease cases) impact response. **h**, Global ozone depletion impact response. **i**, Global respiratory effects (particulate matter less than 2.5 micrometers in diameter) impact response. **j**, Global smog (O_3 emissions) impact response. **k**, Global total energy consumption response. **l**, Global water use response. Underlying data used to create this figure may be found in a data repository at https://doi.org/10.6084/m9.figshare.14390489.v1**.**

Supplementary Figure 10: Environmental impact responses for changing China refined copper imports: China

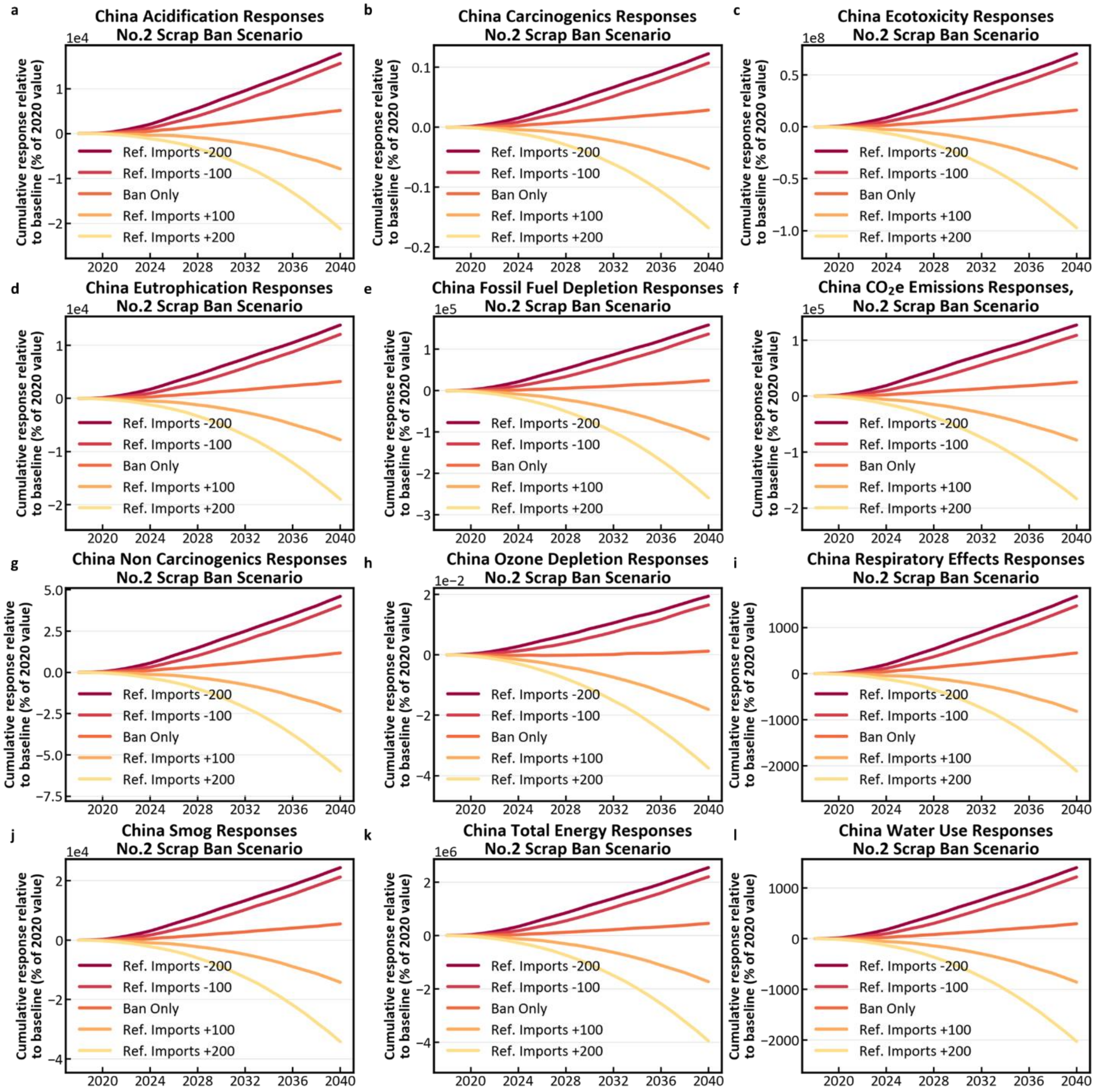

All responses are reported relative to baseline as a percent of the 2020 value. **a**, China SO_2e emissions response. **b**, China carcinogenics human health (disease cases) impact response. **c**, China ecotoxicity (fraction of potentially-affected species integrated over time and volume of freshwater compartment) response. **d**, China N emissions contributing toward eutrophication. **e**, China fossil fuel depletion response. **f**, China CO_2e emissions response. **g**, China non-carcinogenics human health (disease cases) impact response. **h**, China ozone depletion impact response.

i, China respiratory effects (particulate matter less than 2.5 micrometers in diameter) impact response. **j**, China smog (O_3 emissions) impact response. **k**, China total energy consumption response. **l**, China water use response. Underlying data used to create this figure may be found in a data repository at https://doi.org/10.6084/m9.figshare.14390489.v1**.**

Supplementary Figure 11: Environmental impact responses for changing China refined copper imports: Global

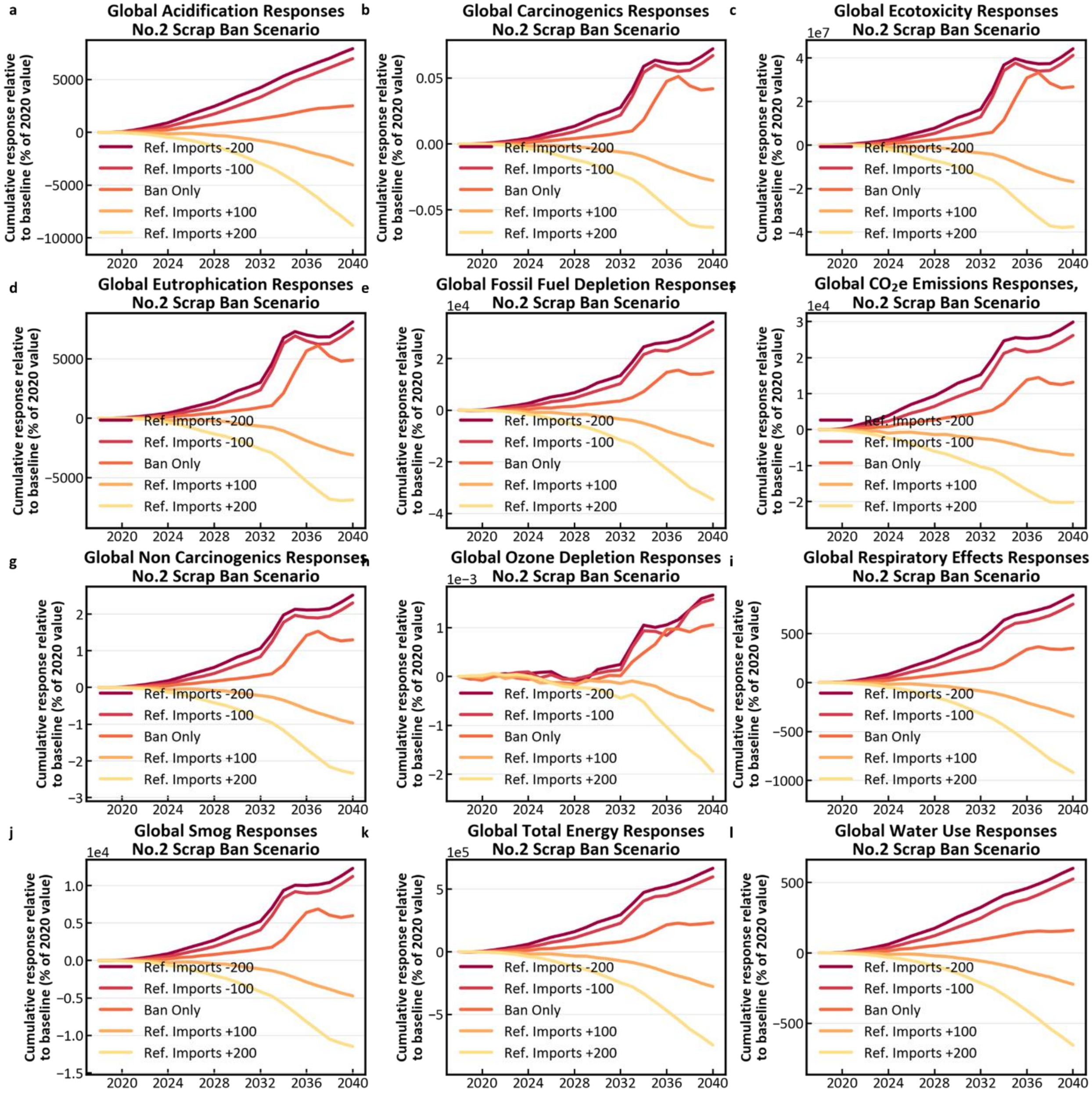

All responses are reported relative to baseline as a percent of the 2020 value. **a**, Global SO_2e emissions response. **b**, Global carcinogenics human health (disease cases) impact response. **c**, Global ecotoxicity (fraction of potentially-affected species integrated over time and volume of freshwater compartment) response. **d**, Global N emissions contributing toward eutrophication. **e**, Global fossil fuel depletion response. **f**, Global CO_2e emissions response. **g**, Global non-carcinogenics human health (disease cases) impact response. **h**, Global ozone depletion impact

response. **i**, Global respiratory effects (particulate matter less than 2.5 micrometers in diameter) impact response. **j**, Global smog (O_3 emissions) impact response. **k**, Global total energy consumption response. **l**, Global water use response. Underlying data used to create this figure may be found in a data repository at https://doi.org/10.6084/m9.figshare.14390489.v1**.**

Supplementary Figure 12: Mean environmental impact responses for supply chain shock relative to baseline

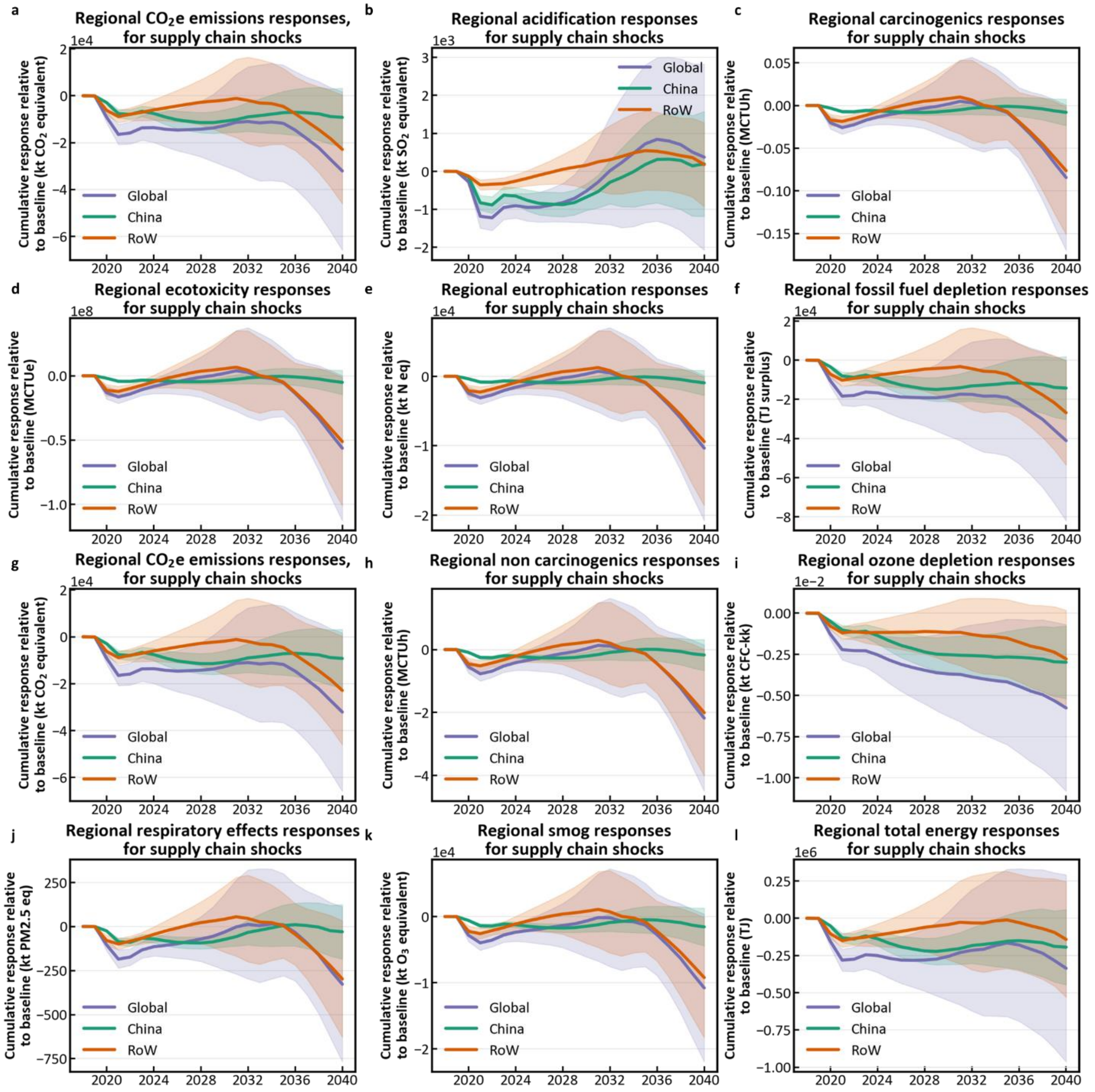

All responses are reported relative to baseline in their original calculated units, and shading represents one standard deviation difference from mean. **a**, Regional CO_2e emissions response. **b**, Regional SO_2e emissions response. **c**, Regional carcinogenics human health (disease cases) impact response. **d**, Regional ecotoxicity (fraction of potentially-affected species integrated over time and volume of freshwater compartment) response. **e**, Regional N emissions contributing toward eutrophication. **f**, Regional fossil fuel depletion response. **g**, Regional non-

carcinogenics human health (disease cases) impact response. **h**, Regional ozone depletion impact response. **i**, Regional respiratory effects (particulate matter less than 2.5 micrometers in diameter) impact response. **j**, Regional smog (O_3 emissions) impact response. **k**, Regional total energy consumption response. **l**, Regional water use response. Underlying data used to create this figure may be found in a data repository at https://doi.org/10.6084/m9.figshare.14390489.v1.

Supplementary Figure 13: Mean environmental impact responses for supply chain shock, as % of 2020 value

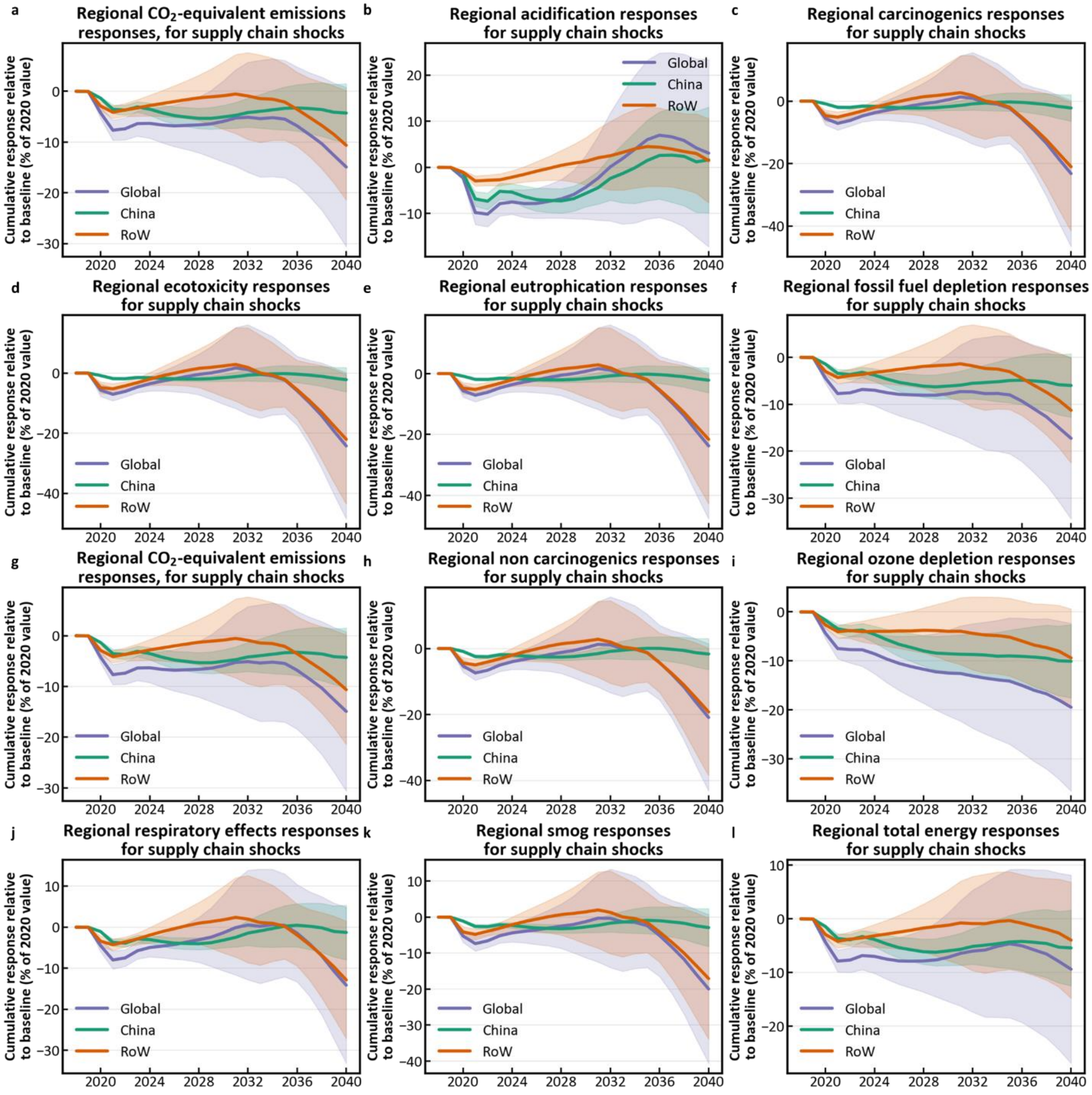

All responses are reported relative to baseline as a percent of 2020 value, and shading represents one standard deviation difference from mean. **a**, Regional CO_2e emissions response. **b**, Regional SO_2e emissions response. **c**, Regional carcinogenics human health (disease cases) impact response. **d**, Regional ecotoxicity (fraction of potentially-affected species integrated over time and volume of freshwater compartment) response. **e**, Regional N emissions contributing toward eutrophication. **f**, Regional fossil fuel depletion response. **g**, Regional non-

carcinogenics human health (disease cases) impact response. **h**, Regional ozone depletion impact response. **i**, Regional respiratory effects (particulate matter less than 2.5 micrometers in diameter) impact response. **j**, Regional smog (O_3 emissions) impact response. **k**, Regional total energy consumption response. **l**, Regional water use response. Underlying data used to create this figure may be found in a data repository at https://doi.org/10.6084/m9.figshare.14390489.v1**.**

Supplementary Data: Impacts of Each Supply Chain Shock

Supplementary Figure 14: Parameter changes for each supply chain shock, with SR included in refinery shocks

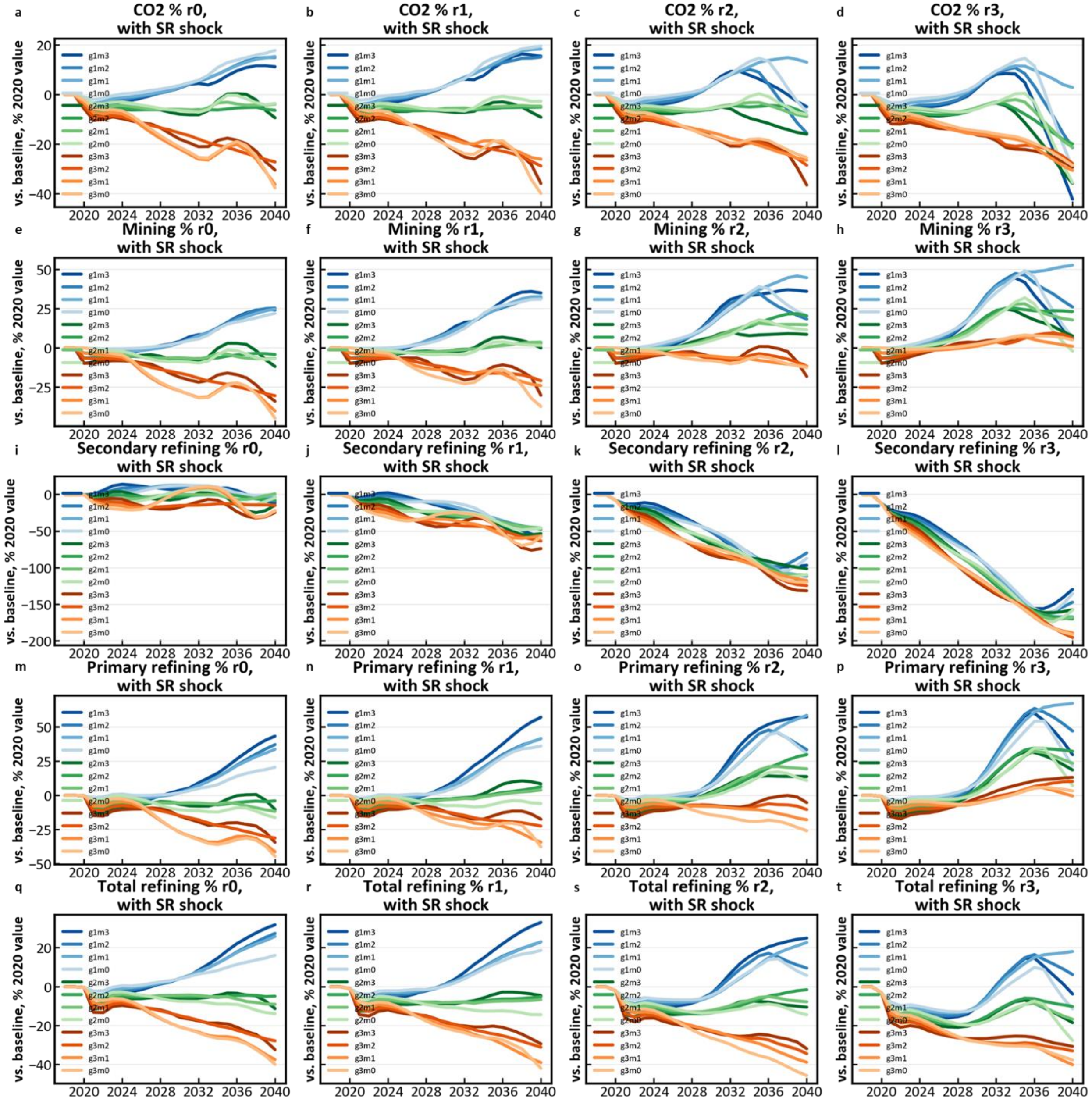

All changes are cumulative, global, and relative to 2020 value. Refinery capacity utilization shocks are zero (r0), 1.14 (r1), 2.29 (r2), and 3.93% (r3) decreases from 2019-2020. SR ratio decreases are zero (r0), 3.46 (r1), 6.93 (r2), and 10.4% (r3) from 2019-2020. GDP per capita decreases are 2 (g1), 4 (g2), and 6%

(g3) from 2019-2020 with proportional rebound. Mining capacity utilization decreases are 0 (m0), 1.31 (m1), 2.62 (m2), and 3.93% (m3) from 2019-2020. Combinations of GDP and mining shocks are shown on each plot, where refinery shocks increase from left to right. **a-d**, CO_2e emissions; **e-h**, mining production; **i-l**, secondary refining production; **m-p**, primary refining production; **q-t**, total refining production.

Underlying data used to create this figure may be found in a data repository at https://doi.org/10.6084/m9.figshare.14390489.v1.

Supplementary Figure 15: Parameter changes for each supply chain shock, with SR excluded from refinery shocks

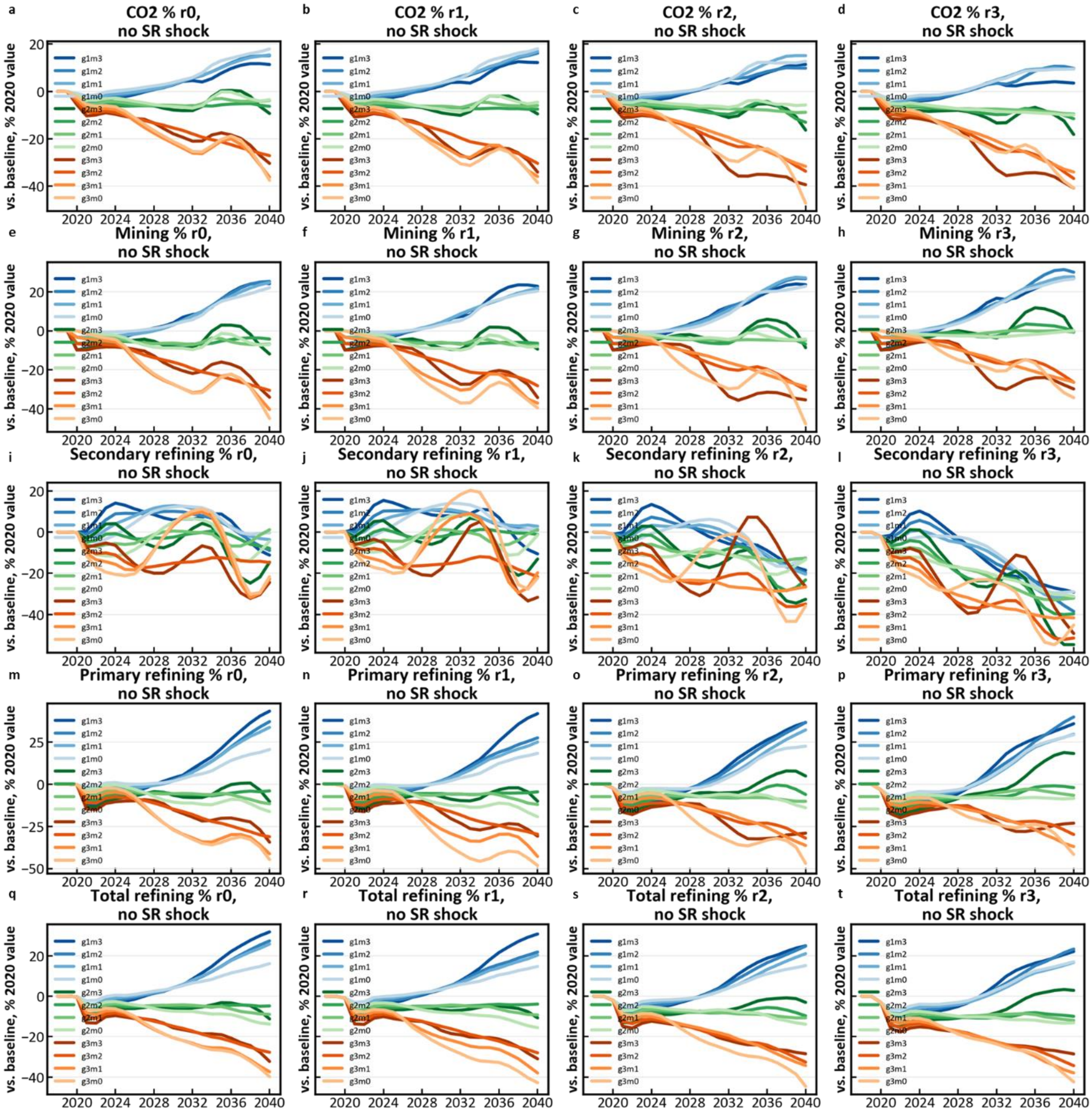

All changes are cumulative, global, and relative to 2020 value. Refinery capacity utilization shocks are zero (r0), 1.14 (r1), 2.29 (r2), and 3.93% (r3) decreases from 2019-2020. SR ratio remains unchanged from baseline. GDP per capita decreases are 2 (g1), 4 (g2), and 6% (g3) from 2019-2020 with proportional rebound. Combinations of GDP and mining shocks are shown on each plot, where refinery

shocks increase from left to right. Mining capacity utilization decreases are 0 (m0), 1.31 (m1), 2.62 (m2), and 3.93% (m3) from 2019-2020. **a-d**, CO_2e emissions; **e-h**, mining production; **i-l**, secondary refining production; **m-p**, primary refining production; **q-t**, total refining production. Underlying data used to create this figure may be found in a data repository at https://doi.org/10.6084/m9.figshare.14390489.v1.

Supplementary Data: Life Cycle Assessment Data

Supplementary Table 11. Regional impact scaling factors for concentrate and SX-EW mines.

		TRACI Scale	Energy Scale	Water Scale
SX-EW Mines	Oceania	1.23	1.75	0.36
	Africa	5.67	4.28	0.47
	Europe	10.56	2.80	0.29
	North America	0.80	2.55	0.28
	China	23.03	9.71	1.31
	Other Asia	23.03	9.71	1.31
	Latin America	0.82	1.64	0.15
Concentrate Mines	Oceania	0.75	1.22	0.84
	Africa	1.55	2.37	0.87
	Europe	0.71	1.24	0.34
	North America	1.11	1.93	0.86
	China	2.34	3.69	1.86
	Other Asia	2.60	4.07	1.89
	Latin America	0.83	1.33	0.40

Supplementary Table 12. Regional environmental impact indicators for concentrate and SX-EW mines, for one kg of Cu.

		Ozone depletion (kg CFC-kk)	Global warming (kg CO2 eq)	Smog (kg O3 eq)	Acidific-ation (kg SO2 eq)	Eutroph-ication (kg N eq)	Carcino-genics (CTUh)	Non carcinog-enics (CTUh)	Respiratory effects (kg PM2.5 eq)	Ecotoxicity (CTUe)	Fossil fuel depletion (MJ surplus)	Total Energy (MJ)	Water use (m3)
SX-EW Mines	Oceania	3.5E-07	4.5E+00	1.9E+00	8.3E-02	8.6E-01	7.3E-06	1.9E-04	1.1E-02	4.6E+03	4.7E+00	7.5E+01	1.8E-02
	Africa	6.9E-07	9.6E+00	2.8E+00	1.3E-01	2.0E+00	1.6E-05	4.2E-04	1.8E-02	1.1E+04	9.3E+00	1.5E+02	1.8E-02
	Europe	2.9E-07	3.1E+00	1.3E+00	5.9E-02	4.0E-01	3.5E-06	8.6E-05	8.4E-03	2.1E+03	3.2E+00	5.8E+01	6.8E-03
	North America	7.8E-07	8.8E+00	4.2E+00	1.8E-01	3.0E+00	2.5E-05	6.5E-04	2.6E-02	1.6E+04	9.3E+00	1.5E+02	1.9E-02
	China	1.1E-06	1.5E+01	6.2E+00	2.7E-01	3.3E+00	2.8E-05	7.3E-04	3.8E-02	1.8E+04	1.3E+01	2.6E+02	3.9E-02
	Other Asia	1.1E-06	1.5E+01	6.2E+00	2.7E-01	3.3E+00	2.8E-05	7.3E-04	3.8E-02	1.8E+04	1.3E+01	2.6E+02	3.9E-02
	Latin America	3.7E-07	4.7E+00	2.1E+00	9.3E-02	1.7E+00	1.4E-05	3.7E-04	1.3E-02	9.1E+03	4.8E+00	8.1E+01	8.3E-03
Concentrate Mines	Oceania	1.1E-07	1.6E+00	7.3E-01	3.4E-02	4.9E-01	4.0E-06	1.1E-04	2.6E-02	2.6E+03	1.7E+00	2.5E+01	6.5E-02
	Africa	2.2E-07	3.4E+00	1.1E+00	5.3E-02	1.1E+00	8.9E-06	2.4E-04	4.1E-02	5.9E+03	3.5E+00	5.0E+01	6.7E-02
	Europe	8.9E-08	1.1E+00	5.3E-01	2.4E-02	2.3E-01	2.0E-06	4.9E-05	1.9E-02	1.2E+03	1.2E+00	1.9E+01	2.5E-02
	North America	2.4E-07	3.1E+00	1.6E+00	7.5E-02	1.7E+00	1.4E-05	3.7E-04	5.8E-02	9.2E+03	3.4E+00	5.1E+01	6.9E-02
	China	3.6E-07	5.4E+00	2.4E+00	1.1E-01	1.9E+00	1.5E-05	4.1E-04	8.7E-02	1.0E+04	4.8E+00	8.3E+01	1.4E-01
	Other Asia	3.6E-07	5.4E+00	2.4E+00	1.1E-01	1.9E+00	1.5E-05	4.1E-04	8.7E-02	1.0E+04	4.8E+00	8.3E+01	1.4E-01
	Latin America	1.2E-07	1.7E+00	8.3E-01	3.8E-02	9.5E-01	7.7E-06	2.1E-04	3.0E-02	5.2E+03	1.8E+00	2.6E+01	3.1E-02

Supplementary Table 13. Constants used to calculate CO_2e emissions, energy consumption, and water use as function of ore grade as described by equation (2) in the main body.

	A	B
Concentrate TRACI	1.58	-0.626
SX-EW TRACI	2.06	-1.208
Concentrate Energy	15.7	-0.573
SX-EW Energy	36.5	-0.351
Concentrate Water	0.0734	-0.094
SX-EW Water	0.0412	-0.340

Supplementary Table 14. Calculated average ore grade for each region considered in this study, used to develop regional impact scaling factors

	Concentrate Ore Grade	SX-EW Ore Grade
Oceania	0.62	0.62
Africa	0.59	1.18
Europe	1.01	4.97
North America	0.40	0.25
China	0.54	2.55
Asia	0.64	2.55
Latin America	0.67	0.43
Average	0.65	0.67

Supplementary Table 15. Direct melt scrap and secondary refining impact indicators.

		Ozone depletion (kg CFC-kk)	Global warming (kg CO2 eq)	Smog (kg O3 eq)	Acidifi-cation (kg SO2 eq)	Eutrop-hication (kg N eq)	Carcin-ogenics (CTUh)	Non carcin-ogenics (CTUh)	Respi-ratory effects (kg PM2.5 eq)	Ecotoxicity (CTUe)	Fossil fuel depletion (MJ surplus)	Total Energy (MJ)	Water use (m3)
Regional Secondary Refining	Oceania	8.63E-08	1.74E-01	9.73E-03	3.84E-03	5.92E-04	1.63E-08	1.32E-07	2.48E-04	4.09E+00	3.50E-01	6.08E+00	1.55E-03
	Africa	1.45E-07	2.55E-01	1.31E-02	1.52E-03	1.14E-03	2.98E-08	2.19E-07	1.72E-04	7.85E+00	6.01E-01	8.68E+00	1.58E-03
	Europe	1.03E-07	1.21E-01	5.35E-03	2.09E-04	3.88E-04	5.49E-09	3.35E-08	5.24E-05	1.32E+00	3.43E-01	4.73E+00	7.14E-04
	North America	2.57E-07	2.45E-01	1.94E-02	1.33E-03	1.75E-03	4.39E-08	2.81E-07	1.92E-04	1.20E+01	6.86E-01	1.01E+01	1.51E-03
	China	1.76E-07	3.16E-01	2.23E-02	2.17E-03	1.61E-03	4.19E-08	2.98E-07	2.96E-04	1.11E+01	6.40E-01	1.09E+01	2.99E-03
	Other Asia	1.76E-07	3.16E-01	2.23E-02	2.17E-03	1.61E-03	4.19E-08	2.98E-07	2.96E-04	1.11E+01	6.40E-01	1.09E+01	2.99E-03
	Latin America	1.02E-07	1.91E-01	1.25E-02	1.23E-03	1.13E-03	2.94E-08	2.15E-07	1.39E-04	7.81E+00	3.79E-01	6.74E+00	8.48E-04
Direct Melt	Low Grade	1.12E-06	1.40E+00	7.97E-02	8.08E-03	3.79E-03	1.50E-07	6.53E-07	1.01E-03	2.35E+01	3.35E+00	3.86E+01	1.19E-02
	Brass	3.11E-07	3.90E-01	2.22E-02	2.25E-03	1.05E-03	4.17E-08	1.82E-07	2.82E-04	6.54E+00	9.33E-01	1.07E+01	3.31E-03
	No2	4.09E-07	5.12E-01	2.91E-02	2.95E-03	1.38E-03	5.48E-08	2.39E-07	3.70E-04	8.58E+00	1.23E+00	1.41E+01	4.34E-03
	No1	2.77E-07	3.46E-01	1.97E-02	2.00E-03	9.37E-04	3.71E-08	1.62E-07	2.51E-04	5.81E+00	8.29E-01	9.54E+00	2.94E-03

Supplementary Table 16. Primary refining impact indicators.

	Ozone depletion (kg CFC-kk)	Global warming (kg CO2 eq)	Smog (kg O3 eq)	Acidif-ication (kg SO2 eq)	Eutrop-hication (kg N eq)	Carcino-genics (CTUh)	Non carcino-genics (CTUh)	Respi-ratory effects (kg PM2.5 eq)	Ecotoxicity (CTUe)	Fossil fuel depletion (MJ surplus)	Total Energy (MJ)	Water use (m3)
Oceania	1.08E-07	2.09E+00	2.88E-01	1.09E+00	1.42E-01	1.34E-06	6.25E-05	4.50E-02	7.18E+02	1.74E+00	3.20E+01	1.88E-02
Africa	1.82E-07	3.06E+00	3.89E-01	4.32E-01	2.74E-01	2.44E-06	1.04E-04	3.14E-02	1.38E+03	2.99E+00	4.56E+01	1.92E-02
Europe	1.30E-07	1.45E+00	1.58E-01	5.92E-02	9.32E-02	4.49E-07	1.59E-05	9.54E-03	2.32E+02	1.71E+00	2.49E+01	8.65E-03
North America	3.22E-07	2.94E+00	5.74E-01	3.78E-01	4.19E-01	3.59E-06	1.33E-04	3.50E-02	2.11E+03	3.41E+00	5.29E+01	1.82E-02
China	2.21E-07	3.79E+00	6.59E-01	6.16E-01	3.85E-01	3.43E-06	1.41E-04	5.39E-02	1.94E+03	3.18E+00	5.75E+01	3.62E-02
Other Asia	2.21E-07	3.79E+00	6.59E-01	6.16E-01	3.85E-01	3.43E-06	1.41E-04	5.39E-02	1.94E+03	3.18E+00	5.75E+01	3.62E-02
Latin America	1.27E-07	2.29E+00	3.70E-01	3.50E-01	2.72E-01	2.41E-06	1.02E-04	2.53E-02	1.37E+03	1.88E+00	3.54E+01	1.03E-02

Supplementary Table 17. Fabrication impact indicators.

	Ozone depletion (kg CFC-kk)	Global warming (kg CO2 eq)	Smog (kg O3 eq)	Acidif-ication (kg SO2 eq)	Eutroph-ication (kg N eq)	Carcin-ogenics (CTUh)	Non carcinogenics (CTUh)	Respiratory effects (kg PM2.5 eq)	Ecotoxicity (CTUe)	Fossil fuel depletion (MJ surplus)	Total (MJ)	Water use (m3)
RoW Metal Working	9.87E-07	3.58E+00	3.64E-01	8.46E-02	1.47E-01	1.37E-06	3.59E-05	1.15E-02	7.83E+02	4.95E+00	6.65E+01	2.22E-02
EU and NAM Metal Working	1.01E-06	3.12E+00	3.35E-01	8.30E-02	1.48E-01	1.36E-06	3.59E-05	1.03E-02	7.82E+02	4.62E+00	6.57E+01	2.29E-02
Global wire drawing	1.82E-07	7.71E-01	7.25E-02	1.57E-02	2.65E-02	2.48E-07	6.29E-06	2.17E-03	1.36E+02	9.56E-01	1.47E+01	6.19E-03

Supplementary Table 18. Impact indicators for producing each alloying element considered in this study.

	Ozone depletion (kg CFC-kk)	Global warming (kg CO2 eq)	Smog (kg O3 eq)	Acidification (kg SO2 eq)	Eutrophication (kg N eq)	Carcinogenics (CTUh)	Non carcinogenics (CTUh)	Respiratory effects (kg PM2.5 eq)	Ecotoxicity (CTUe)	Fossil fuel depletion (MJ surplus)	Total Energy (MJ)	Water use (m3)
Zn	2.37E-07	5.80E+00	6.88E-01	5.36E-02	6.98E-02	7.21E-07	5.21E-05	1.11E-02	3.23E+02	4.10E+00	7.60E+01	1.89E-02
Pb	-1.17E-07	5.08E-02	-2.67E-01	1.90E-02	4.98E-02	7.19E-07	2.53E-05	3.03E-03	2.77E+02	-1.35E+00	-5.06E+00	-5.31E-03
Sn	1.64E-06	1.93E+01	3.40E+00	4.38E-01	9.54E-02	2.55E-06	6.87E-06	1.12E-01	2.00E+02	1.98E+01	3.08E+02	1.36E-01
Ni	1.25E-06	1.18E+01	2.02E+00	1.46E+00	-3.09E-02	-4.95E-08	-2.55E-05	1.08E-01	-3.79E+02	1.32E+01	1.83E+02	-1.11E-01
Al	7.51E-07	1.96E+01	1.22E+00	1.10E-01	6.00E-02	5.53E-06	5.63E-06	2.25E-02	2.45E+02	1.01E+01	2.22E+02	1.38E-02
Mn	2.16E-07	2.80E+00	3.80E-01	1.85E-02	1.97E-02	5.26E-05	1.28E-06	1.71E-02	7.47E+02	2.79E+00	5.53E+01	1.04E-02
Fe	1.39E-08	1.09E-01	1.79E-02	9.79E-04	4.34E-04	6.52E-09	3.09E-08	9.52E-04	8.09E-01	1.36E-01	1.69E+00	6.32E-04

Supplementary Table 19. Regional distribution of each supply chain activity within RoW, where China's fraction is listed for 2017 and permitted to evolve within the model.

	Fabricators	Refineries	SX-EW Mines	Concentrate Mines
Oceania	0	3	0	5
Africa	2	11	24	11
Europe	41	32	1	14
North America	20	12	23	14
Other Asia	32	15	2	9
Latin America	5	27	50	46
China	63	48	0	9

Supplementary Table 20. Regional CO_2e emissions intensity of energy generation over time using data from the U.S. Energy Information Administration's reference case and the countries and regions comprising each region in this study.[22] Country and region emissions intensity of energy generation values were weighted according to their energy consumption by energy-intensive manufacturing. Tables used include "World carbon dioxide intensity of energy use by region" and "Industrial energy consumption by region and sector." Units are tonnes CO_2/billion BTU. Our implementation treated these values as reductions relative to 2018.

	North America	Latin America	Oceania	China	Other Asia	Europe	Africa
EIA Countries or Regions<br><br>Year	United States, Canada	Mexico and Chile, Brazil, Other Non-OECD Americas	Australia and New Zealand	China	India, Other, Middle East, Japan, South Korea, Other Non-OECD Asia	OECD Europe, Russia, Other Non-OECD Europe (Other Europe/Eurasia)	Africa
2018	50.89	40.04	60.34	69.62	61.71	49.77	53.24
2019	50.50	39.83	59.18	68.36	61.03	49.15	53.07
2020	49.58	39.62	58.16	67.19	60.31	48.51	52.78
2021	49.03	39.42	57.41	66.15	60.06	48.31	52.56
2022	48.98	39.20	56.76	65.16	59.83	48.05	52.36
2023	48.86	38.99	56.11	64.25	59.62	47.81	52.17
2024	48.71	38.78	55.49	63.35	59.43	47.56	51.96
2025	48.64	38.58	54.88	62.48	59.25	47.31	51.74
2026	48.50	38.46	53.95	61.39	59.06	46.93	51.34
2027	48.30	38.36	53.01	60.34	58.88	46.54	50.91
2028	48.15	38.26	52.10	59.31	58.72	46.15	50.48
2029	48.03	38.16	51.22	58.31	58.56	45.77	50.04
2030	47.86	38.08	50.37	57.34	58.43	45.40	49.61
2031	47.67	37.94	49.90	56.52	58.12	45.26	49.50
2032	47.51	37.81	49.43	55.72	57.85	45.10	49.42
2033	47.41	37.68	49.01	54.95	57.59	44.95	49.33
2034	47.25	37.57	48.61	54.19	57.35	44.81	49.27
2035	47.09	37.48	48.22	53.47	57.13	44.68	49.20
2036	46.97	37.39	48.00	52.96	56.63	44.50	49.21
2037	46.84	37.32	47.75	52.46	56.15	44.32	49.22
2038	46.70	37.25	47.45	51.97	55.70	44.12	49.24
2039	46.58	37.19	47.22	51.51	55.28	43.96	49.28
2040	46.56	37.14	47.06	51.04	54.88	43.79	49.30

Supplementary Data: Compositional Information for Scrap, Products, Refined Materials

Supplementary Table 21. Upper and lower compositional bounds for each of the raw materials and products used in this study. Alloys are listed in the UNS system, and may be recognized by their form of "C" followed by a 5-digit number and shape of manufacture. Shapes include tube, wire, PSS (plate, sheet, strip), RBS (rod, bar, solid), and cast. These alloys are used as new scrap categories as well. Old scrap is listed using categorical or ISRI grade, and the 8 refined metals considered as alloying elements are grouped. Each of these categories is grouped and materials are listed alphabetically within the given group. This table may be found in a data repository at https://doi.org/10.6084/m9.figshare.14390489.v1.

Supplementary Methods: Scenario Descriptions and Assumptions

Supplementary Table 22. Scenario descriptions. Unalloyed scrap grades include No.1 and No.2 copper scrap, while alloyed scrap grades include yellow brass, leaded yellow brass, red brass, leaded yellow brass, cartridge, manganese bronze, nickel silver, ocean, aluminum bronze, tin bronze, and leaded tin bronze. All scrap and refined copper imports remain constant in the baseline scenario, with changes detailed in Supplementary Table 19. In the baseline scenario and in the absence of COVID-19 related shocks, GDP/capita changes proceed according to IMF projections made prior to any COVID-19 outbreaks, while refinery and mining operations evolve according to their relevant elasticities.

	Scenario	Description
China solid waste import ban scenarios	Alloyed scrap ban	Alloyed scraps are barred from import to China, leading to (25, 50, or 75)% declines in alloyed scrap imports relative to the previous year, starting in 2019. No.1 and No.2 copper scrap imports remain constant. Decline of 50% year-over-year was selected as the mean ban rate, with 25% and 75% included as sensitivities.
	No.2 scrap ban	Scrap requiring refining are barred from import to China, leading to (25, 50, or 75)% declines in No.2 scrap imports relative to the previous year, starting in 2019. No.1 and alloyed copper scrap imports remain constant. Decline of 50% year-over-year was selected as the mean ban rate, with 25 and 75% included as sensitivities.
	<99% Cu scrap ban	Alloyed scraps and scrap requiring refining are barred from import to China, leading to (25, 50, or 75)% declines relative to the previous year, for all scrap imports except No.1 copper scrap, starting in 2019. No.1 scrap imports remain constant. Decline of 50% year-over-year was selected as the mean ban rate, with 25 and 75% included as sensitivities.
Response to	Change in China's refined copper imports	China's refined copper imports change by (-200, -100, 0, 100, 200) kt/year relative to the prior year's value, starting in 2019. We assume China does not become a net exporter of refined copper and negative refined import values are not permitted. In scenarios without refined copper import changes, China's refined copper imports remain constant at the 2018 level.
COVID-19 supply	GDP/capita reduction	Due to the economic disruption associated with COVID-19, changes in GDP/capita relative to the prior year were evaluated in line with Figure 5a in the main body of this work, where the global reduction from 2019-2020 was (2, 4, 6)%, increasing (2.35, 4.7, 7.05)% from 2020-2021. Mean changes were -4% and 4.7% for 2019-2020 and 2020-2021 respectively. All years beyond 2021 use baseline GDP/capita evolution.

Refinery shock	Due to factory closures and shipping restrictions associated with COVID-19, two simultaneous changes in refinery operation occurred, respectively. First, refinery capacity utilization was reduced by (1.14, 2.29, 3.93)% 2019-2020, with mean value 2.29%. Second, shipping restrictions reduced scrap use, causing refinery secondary ratios to decrease (3.46, 6.93, 10.4)% 2019-2020, with 6.93% used as mean. Both these shocks are implemented simultaneously except in Supplementary Figure 15, where the secondary ratio shock was removed to highlight result dependence on this component of the refinery shock over the capacity utilization reduction. For 2021 onward, and the rest of the simulation, these parameters were not constrained and evolve in accordance with the model.
Mine supply shock	Due to the suspension of mining operations due to COVID-19 outbreaks, mining capacity utilization decreased (1.31, 2.62, 3.93)% globally, with mean 2.62%. For 2021 onward, mine capacity utilization changes were not constrained and evolve in accordance with the model.

Assumptions across all scenarios include:

- China's scrap imports are distributed across the 15 scrap grades considered according to their fraction of old scrap generation in RoW in each year.
- China's imports of refined and scrap copper remain constant at 2018 values unless otherwise noted.
- The conversion from sectoral demand to demand by copper shape (tube, RBS, etc.) remains constant across all years, calculated using the mean of 2006-2010 data. This includes determination of the distribution between alloyed and unalloyed copper manufacturing, where unalloyed products account for the majority of refined copper consumption, and consequently this constant distribution determines global refined copper demand.
- China and RoW are not constrained in their refined copper or scrap demand other than that the sum of their refined copper demand must equal that of global. This allows changes in scrap availability to affect the relative refined demand in each region.

- Scrap prices are functions of the quantity available in each year, determined according to the order book formulation resulting from itemized copper scrap import data from China.
- The fraction of each shape comprised by each alloy remains constant across all years, determined by the number of suppliers manufacturing each alloy in each shape in the US, data from CDA.
- China and RoW primary and secondary refineries' CU and SR elasticities to TCRC and No.2 spread are assumed the same as those determined at the global level due to lack of statistical significance at the regional level.
- Baseline mining production is assumed to increase linearly, with slope equal to the average slope of mining production from 2001-2011, as the sharper average increase from 2011-2018 was assumed to be anomalous.
- Regional SX-EW mining environmental impacts were determined using the SimaPro global value for copper from solvent extraction-electrowinning, scaled by the impacts of regional concentrate mining relative to global average concentrate impacts.
- Future regional distribution of mining, refining, and manufacturing within RoW remains constant relative to 2018 (with distribution between China and RoW determined endogenously). Secondary refining regional distribution within RoW was assumed equal to that of primary refining.
- The emissions intensity of energy generation for mines, refineries, and manufacturers undergoes the same change as that of the corresponding region.
- We assume that 55% of mining emissions stem from the electricity required to run operations, with the majority coming from comminution (grinding and milling) of ore. This component's CO_2 intensity decreases in line with regional CO_2 intensity of energy generation. The remaining 45%

is assumed to come from burning primary energy (e.g. coal, natural gas, diesel, heavy oil, or blasting), where we assume CO_2 intensities do not evolve over time.

Supplementary Methods: Near-Term System Response to COVID-19 Shocks

The range of scenarios explored here reflects the uncertainty surrounding the potential market impacts of COVID-19, including cases of supply surplus and deficit. The 2020 projected 4% decrease in GDP per capita produced a year-over-year decrease in refined copper demand of 2.3% (sensitivities gave 0.6% to 4.1%, Figure 5b), well aligned with projections from Roskill (3-4% decrease),[23] ICSG (4% decrease),[24] and S&P Global (2.4% decrease).[25] Using ICSG's January-May 2019-2020 mining capacity utilization decrease of 2.6% (sensitivity 1.3% to 3.9%), the model produced a mining production decrease of 2.3% (sensitivities gave 1.2% increase to 5.7% decrease), which aligns with projections from ICSG (3% decrease)[24,26] and approximately encompasses that of GlobalData (1.9% increase).[27] Following ICSG data, refinery capacity utilization and secondary ratio were set to decrease 2.3% (1.1-3.4%) and 6.9% (3.5-10%) respectively 2019-2020, resulting in refined production increasing 0.43% year over year (sensitivities gave 1.6% increase to 0.75% decrease). Primary refining production then increases 3.1-3.6% while secondary refining production decreases 5.6-14%. ICSG projected 2020 global refining production to remain unchanged from 2019, with a significant decline in secondary refining production due to the disruption of scrap collection and trade.[26]

From these changes across 48 scenarios with GDP alterations, the average 2019-2020 (2020-2021 due to model temporal resolution) change in cathode price was a decrease of 7.2% with standard deviation of 4.9%. Among the 16 scenarios with limited GDP/capita reduction (2.0%), the average 2020-2021 change in cathode price was a decrease of 2.3% with standard deviation 2.7% (not significantly different from zero), where scenarios with moderate to large changes in refining production generated cathode price

changes above zero and reaching as high as 3.2%. Cathode prices are expected to increase up to 10% from 2020-2021 (2021-2022 in this model) due to the global economic recovery and consequent resurgent demand;[25] our model projects a mean increase of 5.4% with standard deviation 7.9%. When scenarios without changes in mining are omitted, this value increases to 8.2% with standard deviation 6.8% among 63 scenarios. Larger price recovery coincides with increased shock size across all parameters.

Copper cathode price remains high, with the post-recovery average price (2022 to 2040) for the mean of our scenarios 2.4% above the 2019 price. These high prices incentivize primary refining and mining production, leading to a redistribution of primary and secondary refining production – a decrease in scrap consumption and increase in concentrate consumption – while suppressing cumulative total refining production (Figure 5c). Larger reductions in GDP per capita limit the cathode price rebound, mitigating this redistribution and the corresponding rebound in CO_2e emissions (Figure 5d). The mean cumulative decrease in CO_2e emissions produced by 2040 is 28 Mt (13% of 2020 value) with standard deviation 36 Mt (17% of 2020 value). With low GDP shocks excluded, this emissions reduction increases to 44 Mt (20% of 2020 value) with standard deviation 25 Mt (12% of 2020 value) among 32 scenarios. Impacts on the material system are shown in Supplementary Figures S14-S15.

Supplementary Methods: Model Evolution Outline

Supplementary Figure 16. Annual model progression with input parameters corresponding to each stage

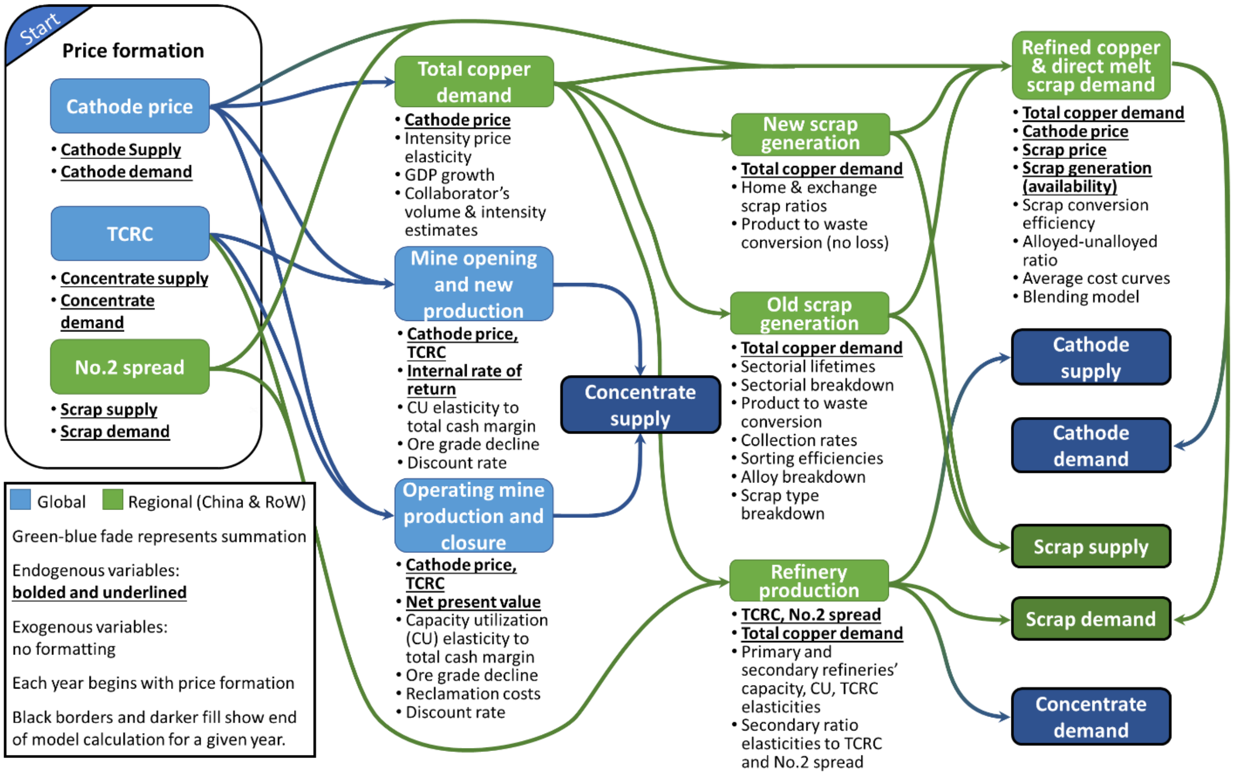

Each year begins with price formation according to cathode, concentrate, and scrap supply and demand. Scrap prices are determined at the regional level. Cathode price and TCRC then inform the total cash margin for evaluating mine opening, closure, and capacity utilization changes. Cathode price induces changes in regional total copper demand, while TCRC and regional No.2 spread cause regional primary and secondary refinery capacity utilization and secondary ratio to change. Old scrap generation is a result of historical total copper demand, material flow analysis, and sectoral and waste distributions. New scrap generation uses that year's total copper demand, home and exchange scrap ratios, and product to waste conversion matrices. Both old and new scrap use the alloy and scrap type breakdowns

described in the Semi-Fabricator Alloy Distribution Framework above. Demand for refined copper and each scrap grade are determined using the Linear Programming Optimization Model described above. Cathode supply is determined by refinery cathode production, cathode demand is determined by the sum of China and RoW refined copper consumption in the Linear Programming Optimization Model, and scrap supply is the sum of post-industrial (new) and post-consumer (old) scrap within each region. Scrap demand is determined by the Linear Programming Optimization Model and remains at the regional level. Concentrate demand is determined by sum of China and RoW concentrate consumption at refineries, and concentrate supply is the sum of all mine production.

References

1 World Copper Factbook 2018. *International Copper Study Group* (2018).

2 ICSG 2018 Statistical Yearbook. *International Copper Study Group* **15** (2018).

3 *Stocks and Flows*, <https://copperalliance.org/about-copper/stocks-and-flows/> (2020).

4 Glöser, S., Soulier, M. & Tercero Espinoza, L. A. Dynamic analysis of global copper flows. Global stocks, postconsumer material flows, recycling indicators, and uncertainty evaluation. *Environmental Science and Technology* **47**, 6564-6572 (2013).

5 International World Copper Council. *Global Semis Production and Demand*, <http://www.coppercouncil.org/iwcc-statistics-and-data> (2020).

6 Copper Development Association. *Copper Alloy Supplier Database*, <https://www.copper.org/resources/suppliers/CDAFabricatorSearch.php> (2020).

7 Copper Development Association. *Copper and Copper Alloys: Compositions, Applications & Properties*, 2004).

8 Jolly, J. L. 2013 Technical Report - The U.S. Copper-base Scrap Industry and its By-products. (2013).

9 CRU Group. *Copper*, <https://www.crugroup.com/analysis/copper/> (2020).

10 S&P Global Market Intelligence. SNL Metals & Mining Data, Subscription. *S&P Global Market Intelligence* (2019).

11 Wood Mackenzie. *Metals and Mining Research*, <https://www.woodmac.com/research/products/metals-and-mining/> (2020).

12 Shanghai Metals Market. *Publication Center*, <https://www.metal.com/> (2020).

13 Shanghai Metals Market. China Copper Market Study. *Shanghai Metals Market* (2019).

14 AMM Scrap Metal Prices. *Fastmarkets AMM*, (2019). Available at <https://www.amm.com/Pricing/ScrapPrices_FullList.html?stubid=14220>.

15 Fastmarkets AMM. *Price Book*, <https://www.metalbulletin.com/> (2020).

16 UN Comtrade Database. *UN Comtrade Online* (2019).

17 Croissant, Y. & Millo, G. Panel data econometrics in R: The plm package. *Journal of statistical software* **27**, 1-43 (2008).

18 Fu, X. *Assessing Byproduct Mining and Metal Recycling as Indicators of Material Criticality* Doctoral thesis, Massachusetts Institute of Technology, (2019).

19 Cooper, J. C. Price elasticity of demand for crude oil: estimates for 23 countries. *OPEC review* **27**, 1-8 (2003).

20 Ryter, J., Fu, X., Bhuwalka, K., Roth, R. & Olivetti, E. A. Emission impacts of China's solid waste import ban and COVID-19 in the copper supply chain - source data. Figshare. identifier [https://doi.org/10.6084/m6089.figshare.14390489.v14390482] (2021).

21 Noshadravan, A., Gaustad, G., Kirchain, R. & Olivetti, E. Operational Strategies for Increasing Secondary Materials in Metals Production Under Uncertainty. *Journal of Sustainable Metallurgy* **3**, 350-361, doi:10.1007/s40831-016-0100-6 (2017).

22 International Energy Outlook 2019. *U.S. Energy Information Administration* (2019).

23 Kinch, D. Metals demand outperforming GDP metrics, copper leads way - Roskill. *S&P Global Market Intelligence*, (2020). Available at <https://www.spglobal.com/platts/en/market-insights/latest-news/metals/082720-metals-demand-outperforming-gdp-metrics-copper-leads-way-roskill>.

24 Selected Data. *International Copper Study Group*, (2020). Available at <https://www.icsg.org/index.php/statistics/selected-data>.

25 Sappor, J. *et al.* COVID-19 impacts — Metals price recovery fragile as recession concerns loom. *S&P Global Market Intelligence*, (2020). Available at

<https://platform.mi.spglobal.com/web/client?auth=inherit&overridecdc=2021&#news/article?id=59221008&KeyProductLinkType=59221023>.
26 The Impact of the COVID-19 Pandemic on World Copper Supply - ICSG Secretariat Briefing Paper. *International Copper Study Group* (2020).
27 Copper production will still grow in 2020, says GlobalData. *MiningJournal.com*, (2020). Available at <https://www.mining-journal.com/copper-news/news/1384164/copper-production-will-still-grow-in-1382020-says-globaldata>.
